# Cytokines as Biomarkers of Pancreatic Ductal Adenocarcinoma: A Systematic Review

**DOI:** 10.1371/journal.pone.0154016

**Published:** 2016-05-12

**Authors:** Yandiswa Yolanda Yako, Deirdré Kruger, Martin Smith, Martin Brand

**Affiliations:** Department of Surgery, Faculty of Health Sciences, University of Witwatersrand, Parktown, Gauteng, South Africa; Centro Nacional de Investigaciones Oncológicas (CNIO), SPAIN

## Abstract

**Objectives:**

A systematic review of the role of cytokines in clinical medicine as diagnostic, prognostic, or predictive biomarkers in pancreatic ductal adenocarcinoma was undertaken.

**Materials and Methods:**

A systematic review was conducted according to the 2009 PRISMA guidelines. PubMed database was searched for all original articles on the topic of interest published until June 2015, and this was supplemented with references cited in relevant articles. Studies were evaluated for risk of bias using the Quality in Prognosis Studies tools.

**Results:**

Forty one cytokines were investigated with relation to pancreatic ductal adenocarcinoma (PDAC) in 65 studies, ten of which were analyzed by more than three studies. Six cytokines (interleukin[IL]-1β, -6, -8, -10, vascular endothelial growth factor, and transforming growth factor) were consistently reported to be increased in PDAC by more than four studies; irrespective of sample type; method of measurement; or statistical analysis model used. When evaluated as part of distinct panels that included CA19-9, IL-1β, -6 and -8 improved the performance of CA19-9 alone in differentiating PDAC from healthy controls. For example, a panel comprising IL-1β, IL-8, and CA 19–9 had a sensitivity of 94.1% vs 85.9%, specificity of 100% vs 96.3%, and area under the curve of 0.984 vs 0.925. The above-mentioned cytokines were associated with the severity of PDAC. IL-2, -6, -10, VEGF, and TGF levels were reported to be altered after patients received therapy or surgery. However, studies did not show any evidence of their ability to predict treatment response.

**Conclusion:**

Our review demonstrates that there is insufficient evidence to support the role of individual cytokines as diagnostic, predictive or prognostic biomarkers for PDAC. However, emerging evidence indicates that a panel of cytokines may be a better tool for discriminating PDAC from other non-malignant pancreatic diseases or healthy individuals.

## Introduction

Pancreatic ductal adenocarcinoma (PDAC) is the most common and aggressive type of pancreatic cancer, accounting for more than 80% of all pancreatic neoplasms diagnosed [1, 2]. It is the fourth leading cause of cancer-related mortality worldwide, accounting for 6% of cancer deaths annually. Various treatment strategies have been introduced over the years, however, with little impact on the 5-year survival rate of 3–5% [3, 4].

Biomarkers for diagnosis, prognosis and predictive response to treatment are necessary to guide patient management and treatment decisions. Specifically, the use of biomarkers to guide therapeutic decisions in non-pancreatic cancers such as colorectal, breast, lung, and prostate cancer is well established. For example, in patients with metastatic colorectal cancer the genetic analysis of the *KRAS* mutation and microsatellite instability is routinely performed to select those that may benefit from therapy with biological agents targeting these mutations [5]. Tumour hormone receptor and human epidermal growth factor receptor-2 (HER2) have been identified as biomarkers for predicting therapeutic response in breast [6] and gastric cancers [7]. With regard to PDAC, serum carbohydrate antigen 19–9 (CA19-9) remains the only routinely used diagnostic and prognostic biomarker. However it has a low sensitivity as approximately 5–10% of the general population do not genetically express the antigenic determinant of CA 19–9 [8], nor is it increased in the early stages of the disease. Furthermore, it exhibits poor prognostic value in patients with localized disease undergoing resection due to falsely elevated levels in the presence of biliary obstruction, and in those receiving chemotherapy [9]. Novel therapeutic targets, therefore, have been investigated and introduced in an attempt to improve survival outcomes.

PDAC is characterized by the presence of dense stromal tissue within the tumour which primarily consists of various inflammatory cell types [10, 11]. Inflammatory cells produce and secrete cytokines, some of which have an immunosuppressive effect, and these include interleukin (IL)-6, IL-10, IL-13, vascular endothelial growth factor (VEGF) and transforming growth factor beta (TGF-β). It is hypothesized that these immunosuppressive cytokines support a favorable environment for the development and progression of PDAC [12, 13]. This review was undertaken to collate the available evidence on alterations of cytokine levels found in PDAC and their implications in diagnosis, prognosis, and prediction of treatment response.

## Materials and Methods

### Data source and Study selection

The review was conducted according to the 2009 PRISMA guidelines [14]. A PubMed search, limited to human clinical studies published in English, was conducted to identify all original articles on the topic of interest published until June 2015, using a combination of search terms as shown in S1 Table. The reference lists of relevant published articles were scanned to supplement the electronic search.

Two investigators (YYY and DK) independently screened retrieved citations by title and abstracts for inclusion into the review. After selecting relevant citations from reviews and meta-analyses, duplicates were removed. Full-text and, where necessary, supplemental materials of publications without abstracts or insufficient information in the abstract were reviewed. Disagreements were solved by consensus or reviewed by a third investigator (MB). Studies were considered eligible if they i) were retrospective or prospective case-control and/or cohort, and ii) investigated the correlation of cytokines with primary PDAC (diagnostic biomarkers), PDAC-related clinical outcomes, such as cancer staging, metastasis, and survival (prognostic biomarkers), and response to treatment regime (predictive biomarkers). Studies were excluded if they did not report on p-values, diagnostic performance (sensitivity and specificity, and area under the curve [AUC]) and/or hazardous ratios with corresponding 95% confidence interval for association analyses. In addition, clinical trial, meta-analyses, reviews, studies with no control group(s), and those that conducted manipulation of cell lines were excluded.

### Data extraction, assessment and synthesis

Two reviewers (YYY and DK) independently extracted data from selected studies on study design, participant characteristics, tumour stage, specific cytokines and their methods of measurement, and the results of any statistical analyses (estimate effect and/or p-values for comparison of cytokine levels between patient and control specimens, and/or specificity and sensitivity values).

#### Quality assessment

Study quality was assessed using the PRISMA Statement [14], excluding items that are used for meta-analysis. Studies were evaluated for their risk of bias using the Quality in Prognosis Studies (QUIPS) tool for prognostic [15] and Quality Assessment of Diagnostic Accuracy Studies (QUADAS) for diagnostic biomarker studies [16, 17]. In this review, we modified the first domain (study participation) of the QUIPS tool to include ethnicity as cytokine concentrations have been shown to be influenced by genetic variations. Retrospective studies were not evaluated for QUIPS items b, c, and e of the second domain (study attrition), as no follow-up is conducted in this type of study design. When evaluating the studies for domain 3 (prognostic factor measurement), we included sample handling and storage as these parameters affect cytokine measurement. Domains 5 (study confounding) and 6 (statistical analysis) were combined and evaluated as a single domain as confounding variables are included in statistical analyses. Risk of bias was graded as high, moderate or low using prompting items. Likewise, we assessed QUADAS-2 ‘domain 1’ (patient selection) using the user’s guide described in the original QUADAS, thus incorporating the spectrum of patients in addition to the signaling questions of QUADAS-2. This allowed us to consider demographics of patients in addition to the study design and selection criteria. Furthermore, we omitted signaling questions 2 and 3 of ‘domain 1’ when assessing the quality of diagnostic studies in a qualification phase of the biomarker development. The discovery and qualification phases of biomarker developmental studies require a case-control design [18]. These questions were replaced as follows: signaling question 2, was a case-control design used? signaling question 3, were selection criteria clearly described? In addition to two signaling questions of ‘domain 2’ (index test), we included ‘item 8’ of the original QUADAS.

## Results

**Fig 1:**
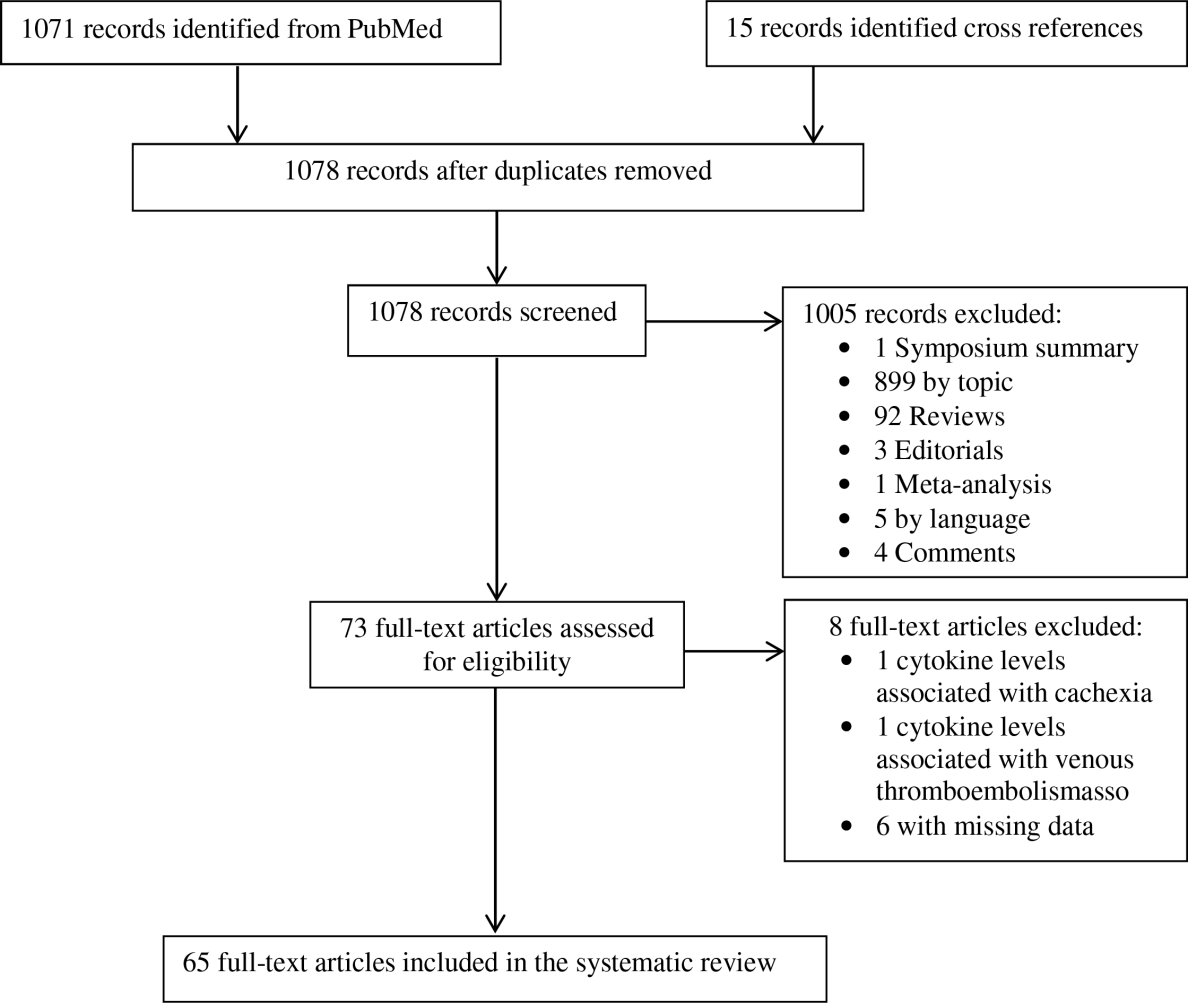
The PRISMA flow diagram illustrating study selection for the systematic review.

A total of 1086 citations were retrieved from PubMed (n = 1071) and references lists (n = 15) as illustrated in Fig 1 and S1 Table. One thousand and thirteen citations were excluded after reviewing titles and abstracts. A further eight of the 73 full-text articles reviewed for eligibility were excluded because i) the concentration of cytokines was associated with related diseases (thromboembolism and cachexia), and ii) p-values and estimate effects were not reported.

### Study characteristics

Sixty five studies analyzing 41 different cytokines were included, and their characteristics are illustrated in S2 Table. Thirty four (52.3%) studies were conducted retrospectively. Sample size varied between 10 and 1564 participants, with the majority of studies comprising mostly men. The majority of studies were conducted in Europe (47.7%), Asia (24.6%), and America (13.8%). More than half of the studies did not specify participant ethnicity, nor were consistent on selection criteria of the study groups. Thirty-six (55.4%) studies compared PDAC patients with healthy individuals, while other studies also included patients with pancreatitis (n = 18) or other hepatobiliary diseases [19]. The age of participants varied between studies from 17 to 93 years old, notably 11 of the studies failed to report an age range [20–26].

The majority of studies measured serum cytokines (n = 38, 58.5%), while others used tissue (n = 14, 21.5%), plasma (n = 12, 18.5%), peripheral blood mononuclear cells (PBMC) (n = 2, 3.1%), pancreatic fluid (n = 1, 1.5%) or whole blood samples (n = 1, 1.5%) (S1 Table). Of the 41 cytokines investigated, 19 were analysed in more than 2 studies: interleukin (IL)-1β, IL-2, IL-6, IL-8, IL-10, IL-12, IL-13, IL-17, IL-23, transforming growth factor-beta1 (TGF-β1), tumor necrosis factor-alpha (TNF-α), macrophage inhibitory cytokine 1 (MIC-1), interferon-gamma (IFN-ɣ), stem cell factor (SCF), macrophage colony-stimulating factor (M-CSF), granulocyte-macrophage colony-stimulating factor (GM-CSF), vascular endothelial growth factor (VEGF), platelet-derived growth factor (PDGF) and epidermal growth factor (EGF). Various methods for measuring cytokines were used of which enzyme-based immunoassays were the most common. Other assays included radioisotopes [27, 28] and electrochemiluminescent tags [29]. Immunohistochemistry was used as an additional method to detect cytokines in tissue samples [21, 23, 24, 30–35]. Alternative methods of measurements included quantitative polymerase chain reaction [24, 25, 31, 33, 36, 37], flow cytometry [38], Northern blot [32] and Western blot [21, 30, 34, 39].

Statistical models varied between studies, and these included t-tests, ANOVA, Wilcoxon rank and Kruskal-Wallis test for descriptive statistical analyses. Approximately, 49% of the studies further investigated the association of cytokine levels with PDAC (regression analysis), and six studies adjusted for confounding variables [34, 40–44]. Additional statistical analyses including diagnostic performance evaluation (sensitivity, specificity, and AUC analyses) was conducted for each cytokine or a combination of cytokines in 11 studies [27, 37, 41, 45–52]. Of these, nine studies compared the diagnostic performance of cytokines to that of CA19-9 [37, 45–52] and three studies to that of carcinoembryonic antigen (CEA) [45, 46, 48]. Diagnostic performance varied according to individual cytokines and to the control group in each study population. Based on the AUC analyses, the combination of cytokines performed better [50–52]. In some studies, the sensitivity seemed to improve but at the expense of specificity [46, 48, 50].

### Quality assessment

**Fig 2:**
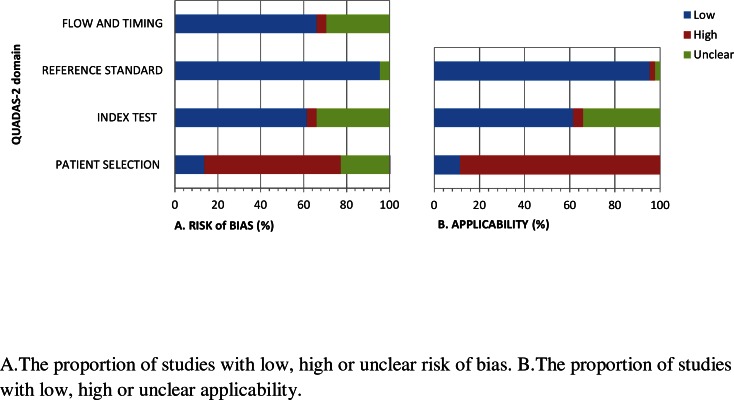
Quality assessment of studies that investigated diagnostic potential of cytokines, according to the QUADAS-2 tool.

Quality assessment was conducted using QUADAS (Fig 2) for diagnostic biomarker studies and the QUIPS tool for prognostic biomarker studies (Table 1).

**Table 1:** Assessment of prognostic biomarker studies for risk of bias using the ‘Quality Assessment in Prognostic studies’ (QUIPS) tool.

Study	Study participants	Study attrition	Prognostic factor measurement	Outcome measurement	Statistical analysis and reporting
Basso et al. 1995 [28]	Moderate	NA	High	Low	High
Fujimoto et al. 1998 [30]	Moderate	Low	High	Low	High
Ikeda et al. 1999 [31]	Moderate	Low	Moderate	Low	High
Wenger et al. 1999 [75]	Moderate	NA	Low	Low	Low
Hashimoto et al. 2001 [56]	Moderate	High	Low	Low	High
Karayiannakis et al. 2001 [79]	Moderate	NA	Low	Low	Moderate
Nagakawa et al. 2002 [85]	Moderate	Low	Low	Low	High
Yue et al. 2002 [22]	Moderate	NA	Low	Low	High
Karayiannakis et al. 2003 [86]	Moderate	Low	Low	Low	High
Ebrahimi et al. 2004 [68]	Moderate	Low	Low	Low	High
Mroczko et al. 2004 [81]	Moderate	NA	Moderate	Low	High
Sears et al. 2004 [76]	Moderate	Low	Low	Low	High
Culhaci et al. 2005 [77]	Moderate	Low	High	Low	High
Mroczko et al. 2005 [45]	Moderate	NA	High	Low	High
Bang et al. 2006 [73]	Moderate	Low	Moderate	Low	High
Bellone et al. 2006 [33]	Moderate	NA	Moderate	Low	High
Lin et al. 2006 [40]	Low	High	Low	Low	Low
Poch et al. 2007 [57]	Moderate	High	Low	Low	High
Groblewska et al. 2007 [46]	Moderate	NA	Low	Low	Moderate
Moses et al. 2009 [58]	Moderate	High	Low	Low	Low
Bellone et al. 2009 [72]	Moderate	Low	Low	Low	High
Talar-Wojnarowska et al. 2009 [70]	Low	NA	Low	Low	Low
Mroczko et al. 2010 [55]	Moderate	High	Low	Low	High
Vizio et al. 2010 [82]	Moderate	Low	Low	Low	High
Baine et al. 2011 [37]	Low	NA	Low	Low	Moderate
Rahbari et al 2011 [83]	Moderate	Low	Low	Low	Moderate
Dima et al. 2012 [61]	Moderate	Low	Low	Low	Low
Sakamoto et al. 2012 [19]	Moderate	High	Low	Low	Moderate
Vizio et al. 2012 [65]	Moderate	Low	Low	Low	Moderate
Ishikawa et al. 2013 [38]	Moderate	Low	Low	Low	High
Schultz et al. 2013 [44]	Moderate	Low	Low	Low	Moderate
Mitsunaga et al. 2013 [29]	Moderate	Low	Low	Low	Moderate
Blogowski et al. 2014 [49]	Moderate	NA	Low	Low	Moderate

NA, not applicable

#### Diagnostic biomarker studies

Forty four (68%) studies evaluated the diagnostic potential of cytokines. The overall result of the QUADAS quality assessment is shown in Fig 2, with specific details in S3 Table. The majority of studies in this category were in the biomarker discovery and qualification phases, investigating cytokines no further than their association with PDAC. Thirteen studies (20%) were in the validation phase as they conducted diagnostic accuracy, and these are indicated by superscript ‘V’ in S3 Table. None of these studies avoided the use of a case-control study design, and were therefore ranked as having “high risk of bias”. Three of 13 studies used a different set of participants for the validation phase [37, 50, 53], while the remaining studies conducted diagnostic accuracy using one set of individuals who participated in the study. Although studies that examined the diagnostic potential of cytokines used a case-control design as per requirement for the qualification phase of biomarker development pipeline [18, 54], the majority of studies failed to report on ethnicity of participants. Similarly, validation studies used a case-control study design instead of consecutively or randomly recruited eligible participants with suspected disease. For this reason, these studies were ranked as having “high risk of bias”. Lifestyle factors such as smoking and alcohol consumption, which are important risk determinants of PDAC were not reported nor evaluated for modifying effects by the majority of diagnostic biomarker studies. We assigned “high risk of bias” to these studies. With regard to the execution of the index test, studies that were assigned “unclear” provided incomplete information on experimental parameters while those ranked as having “high risk of bias” failed to report on this aspect of the study. Studies used computed tomography, endoscopic ultrasound, or histological examination as confirmation modalities of PDAC. Although a few studies compared the diagnostic accuracy of cytokines to that of CA19-9, CA19-9 was not used alone as a gold standard for diagnosis. The index test was interpreted with the knowledge of PDAC diagnosis status in all studies. The majority of studies collected test sample material at the time of diagnosis, and therefore ranked as having “low risk of bias” for ‘domain 4’ (flow and timing).

#### Prognostic biomarker studies

Thirty nine (60%) studies evaluated the prognostic potential of cytokines. The majority of these studies showed a moderate risk of bias for domain 1 (‘study participants’), primarily due to the lack of reporting study population ethnicity. Study attrition is a measure of biasness due to drop-outs in a prospective study [15]. It addresses representation of the experimental participants when there is a loss of individuals due to various reasons during a follow-up period. Eleven studies (16.9%) were conducted retrospectively, and therefore were not assessed for study attrition. Five studies had high risk of bias due to missing data on participants that were lost to follow-up [40], exclusion of patients with missing data [19, 55], and failing to report the duration of follow-up [56–58]. Five (7.7%) studies failed to report on one or more experimental parameters such as sample processing, handling (freezing and thawing cycles) and storage and thus were ranked as having a moderate or high risk of bias for prognostic factor measurement (domain 3), respectively. Moreover, 23 of the 33 studies (69.7%) conducted regression analyses, with 30.8% of them not conducting or conducting incomplete evaluation of potential risk factors. These studies were ranked as having high or moderate risk of bias, respectively.

### Cytokine levels in PDAC patients

Ten of 41 cytokines were investigated in more than three studies, of which six (IL-1β; IL-6, IL-8, IL-10, TGF, and VEGF,) were consistently found to be higher in PDAC patients in more than four studies. These are illustrated in Tables 2 (diagnostic cytokines), 3 (prognostic cytokines), and 4 (predictive cytokines). Forty four studies investigating 10/41 cytokines were diagnostic, and 17 examined both their prognostic and diagnostic potential. The outcomes of individual cytokine analyses are described below.

**Table 2:** Cytokines associated with primary PDAC and are potential diagnostic markers (only cytokines investigated by more than 3 studies are listed).

Cytokine	Sample type	Method of detection	Outcome	Estimate effect and p-values	Country and Population (if specified)	Reference
**IL-1β**						
	Serum	Radioimmunoassay	↑ levels in PDAC patients vs healthy controls.	p < 0.05	Italy	Basso et al. 1995[28]
	Serum	ELISA	↓ levels in PDAC patients vs healthy controls.	p = 0.005	Italy	Bellone et al. 2006 [33]
	Serum	ELISA	NS	NR	Italy (ethnicity not specified)	Fogar et al. 1998 [60]
	Serum	ELISA	↑ levels in pancreatic adenocarcinoma patients vs healthy controls.	p < 0.005	Germany	Poch et al. 2007 [57]
	Serum	Immunoassay	↑ IL-1β in PDAC vs healthy controls.	p < 0.001	China	Zhang et al. 2014 [53]
	Serum	ELISA	↑ levels in PDAC patients vs benign biliary obstruction patients.	p < 0.05	Britain	Shaw et al. 2014 [50]
	Serum	MILLIPLEX High Sensitivity Human Cytokine	NS	p > 0.05	Romania (ethnicity not specified)	Dima et al. 2012 [61]
	PBMC	ELISA	↑ levels in PDAC patients with a 2/2 genotype vs those with 1/2 genotype.	p = 0.046	United Kingdom	Barber et al. 2000 [59]
			↑ levels in PDAC patients with a 2/2 genotype vs those with 1/1 genotype.	p = 0.027		
	Tissue	Immunohistochemistry	↑ staining in PDAC tumoral tissues vs normal tissues.	p < 0.014	Italy	Bellone et al. 2006 [33]
**IL-2**						
	Serum	EIA	↑ IL-2 in untreated PDAC patients vs healthy controls.	p = 0.039	NR	Plate et al. 1999 [62]
	Serum	ELISA	↓ IL-2 in PDAC patients vs healthy controls.	p < 0.005	Germany	Poch et al. 2007 [57]
	Serum	Immunoassay	↑ levels in PDAC patients vs healthy controls.	p < 0.001	China	Zhang et al. 2014 [53]
**IL-6**						
	Serum	ELISA	NS	NR	United Kingdom	Falconer et al. 1994 [69]
	Serum	ELISA	↑ levels in PDAC patients vs healthy controls.	p < 0.01	Italy	Fogar et al. 1998 [60]
	Serum	ELISA	↑ levels in PDAC patients vs healthy and CP controls.	p < 0.01	Japan	Okada et al. 1998 [63]
	Serum	ELISA	↑ levels in PDAC patients vs healthy controls, 0.5 vs 5.2 pg/ml.	IL-6: p = 0.041; sIL-6R: NS, p = 0.093	United Kingdom	Barber et al. 1999 [64]
	Serum	ELISA	↑ levels in pancreatic adenocarcinoma patients vs healthy controls.	3.21 (1.56–7.09) p = 0.004	USA	Ebrahimi et al. 2004 [68]
	Serum	ELISA	↑levels in pancreatic adenocarcinoma patients vs chronic pancreatitis patients.	p < 0.001	Poland (Caucasians)	Talar-Wojnarowska et al. 2009 [70]
			↑levels in patients vs healthy controls.	p < 0.001		
	Serum	MILLIPLEX High Sensitivity Human Cytokine	↑levels in PDAC patients vs healthy controls.	p < 0.01	Romania	Dima et al. 2012 [61]
			↑levels in PDAC patients vs chronic pancreatitis patients.	p < 0.01		
	Serum	Immunoassay	↑ levels in PDAC patients vs healthy controls.	p < 0.001	China	Zhang et al. 2014 [53]
			↑ levels in PDAC patients vs individuals with benign pancreatic diseases patients.	p < 0.001		
	Serum	Meso Scale Discovery technique similar to ELISA	↑ levels in PDAC patients vs healthy controls.	P = 0.001	USA (85.1% Caucasians and 8.1% African-Americans)	Breitbart et al. 2014 [66]
	Serum	ELISA	↑ levels in PDAC patients vs healthy controls.	p < 0.001	Britain	Shaw et al. 2014 [50]
	Serum	Flow cytometry (Multiplex Bead Immunoassay)	↑ levels in PDAC vs healthy controls.	p < 0.01	NR	Komura et al. 2015 [67]
	Plasma	ELISA	↑levels in patients with exocrine pancreatic carcinoma vs healthy controls.	p = 0.01	NR	Wenger et al. 1999 [75]
	Plasma	Bio-plex	↑levels in pancreatic cancer patients vs healthy controls	p < 0.001	United Kingdom	Gabitass et al. 2011 [78]
	Plasma	ELISA	↑levels in PDAC vs normal controls.	p < 0.001	Italy	Vizio et al. 2012 [65]
	Plasma	ELISA	levels increased with increasing PDAC stage.	p < 0.0001	80.1% Denmark; 19.9% Germany	Schultz et al. 2013 [44]
	Plasma	Not applicable	↑ levels in PDAC patients vs individuals free of cancer.	p = 0.002	USA (Caucasians and African-Americans)	Bao et al. 2013 [43]
	Plasma	ELISA	↑levels in PDAC patients vs healthy controls.		Poland	Blogowski et al. 2014 [49]
	Pancreatic juice	EIA	↑levels in patients with pancreatic cancer vs individuals with normal pancreas.	p < 0.001	American	Noh et al. 2006 [41]
	Tissue	Immunohistochemistry; quantitative PCR	↑mRNA levels in pancreatic carcinoma tissues (median factor = 62.4) vs tumor-free tissues.	p < 0.001	Italy	Bellone et al. 2006 [33]
**IL-8**						
	Serum	ELISA	↑ levels in pancreatic carcinoma patients vs healthy controls.	p < 0.0001	USA	Ebrahimi et al. 2004 [68]
	Serum	ELISA	↑ levels in pancreatic carcinoma patients vs healthy controls.	p < 0.0001	Italy	Bellone et al. 2006 [33]
	Serum	ELISA	↑levels in pancreatic cancer vs chronic pancreatitic patients.	p = 0.002	China	Chen et al. 2012 [71]
			↑levels in pancreatic cancer vs acute pancreatitic patients.	p = 0.041		
			↑levels in pancreatic cancer vs gastric carcinoma patients.	p = 0.025		
			↑levels in pancreatic cancer vs colorectal carcinoma patients.	p = 0.032		
			↑levels in pancreatic cancer vs hepatocellular carcinoma patients.	p = 0.016		
	Serum	Immunoassay	↑ levels in PDAC patients vs healthy control.	p < 0.001	China	Zhang et al. 2014 [53]
			↓ levels in PDAC patients compared vs patients with a benign disease.	p = 0.028		
	Serum	ELISA	↑ levels in PDAC patients with biliary obstruction vs individuals with benign biliary obstruction and chronic pancreatitis.	p < 0.05	Britain	Shaw et al. 2014 [50]
	Serum	Flow cytometry (Multiplex Bead Immunoassay)	Levels relatively high in patients vs healthy controls.	p = NS	NR	Komura et al. 2015 [67]
	Plasma	Antibody suspension bead array	↓ levels in PDAC patients vs patients with pancreatitis and benign hepatobiliary diseases.	p < 0.05	Japanese	Sakamoto et al. 2012 [19]
	Plasma	ELISA	↑ levels in PDAC patients vs healthy controls.	p < 0.03	Poland	Blogowski et al. 2014 [49]
			↑ levels in PDAC patients vs patients with other pancreatic malignancies.	p = 0.05		
	Tissue	Immunohistochemistry; quantitative PCR	↑ mRNA levels in pancreatic carcinoma specimens.	p < 0.001	Italy	Bellone et al. 2006 [33]
			IL-8 detected in few tumoral tissues (7/41 vs. 6/9) by immunohistochemical staining.	p = 0.006		
	Tissue	Western Blot Analysis	↑ mRNA levels in PDAC tissues vs corresponding normal tissues.	p < 0.05	Germany	Frick et al. 2008 [34]
	Tissue	Quantitative PCR Immunohistochemistry	Expressed in 55.6% of pancreatic cancer specimen vs 25.9% non-cancer tissues.	p < 0.01	China	Chen et al. 2012 [71]
			Positive immunostaining in patients.	p = 0.016		
	Pancreatic juice	2-site chemiluminescent immunometric assay	↑ levels in pancreatic cancer patients vs individuals with normal pancreas.	p < 0.001	American	Noh et al. 2006 [41]
			↑ levels in pancreatic cancer patients vs chronic pancreatitis.	p < 0.01		
**IL-10**						
	Serum	ELISA	↑ levels in pancreatic carcinoma patients vs healthy controls.	p = 0.001	USA	Ebrahimi et al. 2004 [68]
	Serum	EIA	↓ levels of IL-10 protein complex in PDAC patients vs healthy controls.	p = 0.037	NR	Plate et al. 1999[62]
	Serum	ELISA	↑ levels in PDAC patients vs healthy controls.	p < 0.0001	Germany	von Bernstorff et al. 2001 [74]
	Serum	ELISA	↑ levels in pancreatic carcinoma patients vs healthy controls.	p = 0.04	Italy	Bellone et al. 2006 [33]
	Serum	ELISA	↑ levels in pancreatic cancer patients vs healthy controls.	p < 0.05	Germany	Poch et al. 2007 [57]
	Serum	MILLIPLEX High Sensitivity Human Cytokine	↑levels in PDAC patients vs healthy controls.	p < 0.001	Romania	Dima et al. 2012 [61]
	Serum	Meso Scale Discovery technique similar to ELISA	↑levels in PDAC patients vs healthy controls.	P = 0.02	America (85.1% Caucasians and 8.1% African-Americans)	Breitbart et al. 2014 [66]
	Serum	Immunoassay	↑levels in PDAC patients vs healthy controls.	p < 0.001	China	Zhang et al. 2014 [53]
			↓levels in PDAC patients vs those of patients with a benign disease.	p < 0.001		
	Plasma	ELISA	↑levels in patients with exocrine pancreatic carcinoma vs healthy controls.	p = 0.03	NR	Wenger et al. 1999 [75]
	Plasma	Bio-plex	↑levels in pancreatic cancer patients vs healthy controls.	p = 0.001	United Kingdom	Gabitass et al. 2011 [78]
	Plasma	ELISA	↑levels in PDAC patients vs healthy controls.	p < 0.0002	Poland	Blogowski et al. 2014 [49]
	Tissue	Quantitative PCR	↑mRNAs levels in PDAC patients.	p < 0.001	Italy	Bellone et al. 2006 [33]
	PBMC	ELISA	↑levels in PDAC patients vs normal controls.	P = 0.023	Italy	Bellone et al. 2009 [72]
**TGF**						
	Serum	ELISA	↑ TGF-β1 and 2 levels in PDAC patients vs healthy and benign-diseased controls.	p < 0.0001	Germany	von Bernstorff et al. 2001[74]
	Serum	ELISA	↑ TGF-β1 levels detected in cancer patients.	p < 0.0001	Italy	Bellone et al. 2006 [33]
	Serum	ELISA	↑ TGF-β1 levels in patients.	p < 0.005	Germany	Poch et al. 2007 [57]
	Serum	ELISA	↑ TGF-β2 levels in cancer patients.	p < 0.0001	Italy	Bellone et al. 2006 [33]
	Serum	Meso Scale Discovery technique similar to ELISA	↓ TGF-β levels in cancer patients.	p = 0.02	America (85.1% Caucasians and 8.1% African-Americans)	Breitbart et al. 2014 [66]
	Plasma	ELISA	↑levels in pancreatic adenocarcinoma patients vs normal controls.	p = 0.003	Italy	Vizio et al. 2012 [65]
	Tissue	Immunohistochemistry	↑ TGF-β1 positive cells in PDAC tissue samples.	p < 0.01	China	Yue et al. 2002 [22]
			↑ TGF-β1 positive cells in patients with lymph node metastasis.	p < 0.05		
			↑over-expression in worse differentiated cancer cells.	p < 0.05		
	Tissue	Immunohistochemistry and quantitative PDACR	↑expression of TGF-β1, TGF-β3 and TGF-β2 PDAC tissue samples.	p < 0.001	Italy	Bellone et al. 2006 [33]
			+ staining of TGF-β2	p = 0.03		
			TGF-β3 in tumoral tissues.	p = 0.01		
	Pancreatic fluid	ELISA	↑levels in patients with pancreatic cancer vs individuals with normal pancreas.	p ≤ 0.03	USA	Noh et al. 2006 [41]
	Urine	radioimmunoassay	TGF-α levels not different in PDAC patients vs healthy controls.	NR	Taiwan	Chuang et al. 1994[27]
TNF-α						
	Serum	ELISA	TNF-α was not detected.	NR	United Kingdom	Falconer et al. 1994 [69]
	Serum	ELISA	↑levels in patients compared to healthy controls.	p < 0.05	Germany	Poch et al. 2007 [57]
	Serum	MILLIPLEX High Sensitivity Human Cytokine	↑levels in PDAC patients vs healthy controls.	p = 0.033	Romania	Dima et al. 2012 [61]
	Serum	Immunoassay	↑levels in PDAC vs healthy controls.	p < 0.001	China	Zhang et al. 2014 [53]
			↓levels in PDAC vs patients with a benign disease.	p < 0.001		
	Plasma	ELISA	NS	p = 0.17	NR	Wenger et al. 1999 [75]
	Plasma	Bio-plex	NS	p = 0.67	United Kingdom	Gabitass et al. 2011 [78]
	Plasma	ELISA	↑levels in PDAC patients vs healthy controls.	p = 0.01	Poland	Blogowski et al. 2014 [49]
			↑levels in PDAC patients vs patients with other pancreatic malignancies.	p = 0.03		
**MIC-1**						
	Serum	ELISA	↑levels in PDAC patients.	p < 0.05.	Australia	Koopmann et al. 2004 [51]
	Serum	ELISA	MIC-1 was an independent predictor of PDAC	AUC (cancer patients vs healthy controls = 0.99 (0.86–1.00), p = 0.003.	Australia	Koopmann et al. 2006 [80]
	Serum	ELISA	↑levels in PDAC patients vs individuals with benign pancreatic disease; biliary diseases; healthy controls.	p < 0.05	Turkey	Ӧzkan et al. 2011 [47]
	PBMC	Quantitative RT-PCR	↓expression levels in early and late PDAC patients vs CP patients.	p = 0.044	America (169 Caucasians, 5 African-Americans, 1 Asian, 2 unknown)	Baine et al. 2011 [37]
	Plasma	ELISA	↑levels in PDAC patients vs healthy controls.	OR (95% CI) at cut-off > 2.3 ng/ml: PDAC vs HC = 2.7 (0.97–7.4) p = 0.056; Stage 1/2 PDAC vs HC:6 (1.9–18.2) p = 0.0018; Stage 3/4 PDAC vs HC = 4.8 (1.6–14.5) p = 0.005.		Kaur et al. 2013 [52]
			↑levels in pancreatic cancer patients vs CP patients.	PDAC vs CP = 5.8 (1.8–18.4) p = 0.0028; Stage 1/2 PDAC vs CP = 11.5 (3.4–39) p < 0.0001; Stage 3/4 PDAC vs CP = 12.8 (2.6–62.2) p = 0.0015		
**M-CSF**						
	Serum	ELISA	↑levels in PDAC patients vs healthy controls and patients with pancreatitis.	p < 0.05	Poland	Mroczko et al. 2005 [45]
	Serum	ELISA	↑levels higher in PDAC patients vs healthy controls.	p < 0.05	Poland	Groblewska et al. 2007 [46]
	Serum	ELISA	↑levels in PDAC patients vs healthy controls.	p < 0.001.	Greece	Vasiliades et al. 2012 [48]
**VEGF**						
	Serum	ELISA	↑VEGF in pancreatic cancer patients vs healthy controls.	p < 0.05.	Korea	Bang et al. 2006 [73]
	Serum	ELISA	↑VEGF in PDAC patients vs healthy controls.	p< 0.001	Taiwan	Chang et al. 2008 [84]
	Serum	ELISA; multiplex protein array	↑VEGF in patients with primary PDAC vs healthy controls.	p < 0.05	Germany	Rahbari et al. 2011 [83]
	Plasma	ELISA	↑VEGF-A levels in PDAC patients vs normal controls.	p< 0.005	Italy	Vizio et al. 2010 [82]
	Plasma	Bio-plex	NS levels between PDAC patients and healthy controls.	p = 0.068	United Kingdom	Gabitass et al. 2011 [78]
			↑ levels in PDAC patients vs those with esophagus and gastric cancers.	p< 0.001		
	Plasma	Antibody suspension bead array	↓levels in PDAC patients vs patients with pancreatitis and benign hepatobiliary diseases.	p < 0.05	Japan	Sakamoto et al. 2012 [19]
	Tissue	Quantitative RT-PCR; immunohistochemistry	+ staining in 67.5% of carcinoma tissues.	p = 0.006	Japan	Ikeda et al. 1999 [31]
	Tissue	Northern blot	↑expression in 55.6% of cancer samples.	p < 0.01	NR	Itakura et al. 2000 [32]

CI, confidence interval; CP, chronic pancreatitis; ELISA, enzyme-linked immunosorbent assay; HR, hazard ratio; IL, interleukin; M-CSF, macrophage colony-stimulating factor; MIC-1, macrophage inhibitory cytokine 1; NR, not reported; NS, non-significant; PBMC, peripheral blood mononuclear cell; PDAC, pancreatic ductal adenocarcinoma; RT-PCR, reverse transcription-polymerase chain reaction; TGF-α, transforming growth factor-alpha; TGF-β, transforming growth factor-beta; TNF, tumor necrosis factor; UICC, Union for International Cancer Control; VEGF, vascular endothelial growth factor.

**Table 3:** Cytokines associated with the disease severity and could play a prognostic role in PDAC (only cytokines investigated by more than 3 studies are listed).

Cytokine	Sample type	Method of detection	Outcome	Estimate effect and p-values	Country and Population (if specified)	Reference
IL-1β						
	Serum	Radioimmunoassay	↑ levels in PDAC patients with metastasis vs those without.	p < 0.01	Italy	Basso et al. 1995 [28]
	Tissue	Quantitative PCR	↓ protein expression associated with shorter survival.	HR (95% CI): 3.41 (1.44–32.66) p < 0.015	Italy	Bellone et al. 2006 [33]
	Serum	Electro-chemiluminescence assay	↑ levels identified as independent predictors of poor overall survival (OS). IL-6^High^/ IL-1β ^High^ group revealed higher risks for death and tumour progression.	HR: 1.88 (1.01–3.45) p = 0.048	China	Mitsunaga et al. 2013 [29]
**IL-2**						
	Whole blood	Flow cytometry	↑ levels in PDAC patients after adoptive T-cell therapy not associated with overall survival.	HR (95% CI) = 1.100 (0.548–2.207) p = 0.789	Japan	Ishikawa et al. 2013 [38]
**IL-6**						
	Serum	ELISA	↑ levels associated with decreased survival.	3.21 (1.56–7.09) p = 0.004	USA	Ebrahimi et al. 2004 [68]
	Serum	ELISA	↑ levels in patients with cachexia vs those without.	p < 0.04	Germany	Martignoni et al. 2005 [36]
	Serum	ELISA	↓ levels in PDAC patients with locally advanced tumors (UICC stages II and III).	UICC stage I vs UICC stage II and III: p = 0.0001.	Italy	Bellone et al. 2006 [33]
			Correlation with longer survival.	p = 0.03.		
	Serum	ELISA	Marginally elevated levels in cachectic pancreatic cancer patients.	p = 0.057	Britain	Moses et al. 2009 [58]
	Serum	ELISA	↑levels associated with tumor size	p < 0.01	Poland (Caucasians)	Talar-Wojnarowska et al. 2009 [70]
	Serum	ELISA	↑ levels in patients with advanced cancer.	p < 0.001.	Poland	Mroczko et al. 2010 [55]
	Serum	Electro-chemiluminescence assay	↑ levels were identified as independent predictors of poor overall survival and short progression-free survival.	HR (overall survival): 2.10 (1.19–3.74), p = 0.011. HR (progression-free survival): 2.32 (1.33–4.07), p = 0.003.	China	Mitsunaga et al. 2013 [29]
			The IL-6^High^/ IL-1β^High^ group revealed higher risks for death and tumour progression.	Overall survival: p < 0.001. Progression-free survival: p < 0.001.		
	Plasma	ELISA	↓levels in PDAC patients at an early disease stage.	p = 0.008	Italy	Vizio et al. 2012 [65]
			Levels not associated with patient survival.	Hazard ratio (95% CI) = 1.002 (0.998–1.007) p = 0.246.		
	Plasma	ELISA	levels increased with increasing PDAC stage.	p < 0.0001	80.1% Denmark; 19.9% Germany	Schultz et al. 2013 [44]
	Tissue	Quantitative PCR	↑ mRNA levels in PDAC patient with cachexia.	p < 0.01	Germany	Martignoni et al. 2005 [36]
**IL-8**						
	Serum	MILLIPLEX High Sensitivity Human Cytokine	IL-8 levels < 9.27 pg/mL associated with longer survival.	p < 0.01	Romania	Dima et al. 2012 [61]
	Plasma	Antibody suspension bead array	↑ levels in metastasis-positive group.	p = 0.024.	Japanese	Sakamoto et al. 2012 [19]
	Plasma	ELISA	↑ levels in locally advanced and metastatic disease.	Beta coefficient (95% CI) = 0.71 (0.61–0.80) p = 0.0008.	Poland	Blogowski et al. 2014 [49]
**IL-10**						
	Serum	ELISA	↑ levels in cancer patients with metastatic tumors (UICC stage IV).	p = 0.008	Italy	Bellone et al. 2006 [33]
	Whole blood	Flow cytometry	↑ levels in PDAC patients not associated with overall survival.	HR (95% CI) = 0.970 (0.474–1.982) p = 0.933.	Japan	Ishikawa et al. 2013 [38]
	Plasma	ELISA	↑levels associated with tumor size.	p = 0.04	NR	Wenger et al. 1999 [75]
	Tissue	Immunohistochemistry	Strong IL-10 staining in patients with stage IV.	p = 0.001	Italy	Bellone et al. 2006 [33]
**TGF-β1**						
	Serum	ELISA	↑ TGF-β1 levels with increasing risk of death from pancreatic cancer.	OR = 2.5 (0.9–6.9), trend p = 0.04.	Japan	Lin et al. 2006 [40]
	Serum	ELISA	↓ levels in patients with locally advanced tumors (UICC stages II and III) vs metastatic tumors (UICC stages IV).	p = 0.004	Italy	Bellone et al. 2006 [33]
	Serum	ELISA	↑ levels in patients with stage IV vs stage I-III tumours.	p < 0.05	Germany	Poch et al. 2007 [57]
	Serum	ELISA	↓ levels associated with longer survival period.	p = 0.02	Italy	Bellone et al. 2006 [33]
	Plasma	ELISA	↑levels in patients vs controls.	p = 0.003	Italy	Vizio et al. 2012 [65]
			↓levels after chemotherapy.	p = 0.032		
	Plasma	ELISA	↑ levels in PDAC patients, but not different between early and late disease stage.	p = 0.431	Italy	Vizio et al. 2012 [65]
			↑ levels in PDAC patients associated with shorter survival.	HR (95% CI) = 1.050 (1.021–1.079) p = 0.001.		
	Tissue	Immunohistochemistry	Expression of TGF-β in tumours associated with longer patient survival.	p = 0.039	Japan	Hashimoto et al. 2001[56]
	Tissue	Immunohistochemistry	↑ TGF-β1 positive cells in patients with lymph node metastasis.	p < 0.05	China	Yue et al. 2002 [22]
			↑ over-expression in worse differentiated cancer cells.	p < 0.05		
	Biopsy obtained through fine-needle aspiration	Immunohistochemistry	+ staining in lower-grade tumours but not statistically different.	NS	USA	Sears et al. 2004 [76]
	Tissue	Immunohistochemistry	+ staining of TGF-β1 in 22.2% tumor tissues and 15.9% weakly positive.	NR	Turkey	Culhaci et al. 2005[77]
			Protein expression not related to patient survival.	NR		
**TNF-α**						
	Serum	ELISA	TNF-α levels were detectable mostly in patients with metastatic disease.	p < 0.01	Greece	Karayiannakis et al. 2001[79]
	Serum	MILLIPLEX High Sensitivity Human Cytokine	TNF-α < 2.45 pg/mL associated with longer survival.	p < 0.01	Romania	Dima et al. 2012 [61]
	Plasma	ELISA	↑levels associated with tumor size.	p = 0.02	NR	Wenger et al. 1999 [75]
	Whole blood	Flow cytometry	↑ levels in PDAC patients not associated with overall survival.	HR (95% CI) = 0.905 (0.451–1.816) p = 0.779	Japan	Ishikawa et al. 2013 [38]
**MIC-1**						
	PBMC	Quantitative RT-PCR	Expression levels increased with cancer progression, but not significantly.	p > 0.05	America (169 Caucasians, 5 African-Americans, 1 Asian, 2 unknown)	Baine et al. 2011 [37]
**M-CSF**						
	Serum	ELISA	↑ levels in PDAC patients with advanced tumour stage.	p < 0.05	Australian	Mroczko et al. 2004 [81]
	Serum	ELISA	↑ levels in PDAC patients with advanced tumour stage IV vs stage III.	NS	Poland	Mroczko et al. 2005 [45]
	Serum	ELISA	↑ levels in patients with non-resectable tumors.	p < 0.05	Poland	Groblewska et al. 2007 [46]
			↓ levels associated with longer patient survival.	p = 0.024		
**VEGF**						
	Serum	ELISA	↑ levels in PDAC patients with lymph node.	p = 0.03	Greece	Karayiannakis et al. 2003 [86]
			↑ levels in PDAC patients with distant metastasis.	p = 0.001		
	Serum	ELISA	↑ VEGF/sVEGF-R1 were associated with shorter patient survival.	HR (95% CI): 1.032 (1.007–1.056) p = 0.01	Taiwan	Chang et al. [84]
	Plasma	ELISA	↑VEGF-A levels in PDAC patients correlated with poor prognosis.	p< 0.005	Italy	Vizio et al. 2010 [82]
	Tissue	Immunohistochemistry	+ staining associated with G3 histological grading,	p = 0.0058	Japan	Ikeda et al. 1999 [31]
			+ staining associated with shorter patient survival (7.5 months).	p = 0.048		
	Tissue	Immunohistochemistry	+ staining in 59.4% of patients and found in 66.7% of patients with liver metastasis.	NS (no p-value provided).	Japan	Nagakawa et al. 2002 [85]
	Tissue	RT-PCR, Western blot, Immunohistochemistry	+ staining associated with microvessel count.	p = 0.002	Japan	Fujimoto et al. 1998 [30]
			Microvessel count associated with advanced PDAC.	p = 0.025		

BEV+CAPE+RT treatment; bevacizumab+capecitabine+radiotherapy; CI, confidence interval; CP, chronic pancreatitis, ELISA, enzyme-linked immunosorbent assay; GEM, gemcitabine; HR, hazard ratio; IL, interleukin; M-CSF, macrophage colony-stimulating factor; MIC-1, macrophage inhibitory cytokine 1; NR, not reported; NS, non-significant; PBMC, peripheral blood mononuclear cell; PDAC, pancreatic ductal adenocarcinoma; RT-PCR, reverse transcription-polymerase chain reaction; TGF-α, transforming growth factor-alpha; TGF-β, transforming growth factor-beta; TNF, tumor necrosis factor; UICC, Union for International Cancer Control; VEGF, vascular endothelial growth factor.

**Table 4:** Cytokines associated with the response to treatment regime and could play a predictive role in PDAC are likely to have a predictive value (only cytokines investigated by more than 3 studies are listed).

Cytokine	Sample type	Method of detection	Outcome	Estimate effect and p-values	Country and Population (if specified)	Reference
**IL-2**	Whole blood	Flow cytometry	↑ levels in patients after adoptive T-cell therapy.	p = 0.0373	Japan	Ishikawa et al. 2013 [38]
**IL-6**	Plasma	ELISA	↓levels after GEM treatment	p = 0.001	Italy	Vizio et al. 2012 [65]
			↓levels after BEV+CAPE+RT treatment.	p = 0.028		
			Levels not statistically different between responders and non-responders	p = 0.178		
**IL-10**	Serum	ELISA	↓ levels in PDAC patients after 28 days of gemcitabine and cisplatin combination chemotherapy.	NS	Korea	Bang et al. 2006 [73]
	PBMC	ELISA	↓ levels in PDAC patients after radical tumour resection.	p = 0.04	Italy	Bellone et al. 2009 [72]
			↓ spontaneous IL-10 levels at time 4 after combined chemotherapy.	p = 0.018		
			↓ lipopolysaccharide-induced IL-10 levels at time 4 after combined chemotherapy.	p = 0.047		
	Whole blood	Flow cytometry	↑ levels in patients after adoptive T-cell therapy.	NS, p = 0.9314	Japan	Ishikawa et al. 2013 [38]
**TGF-β1**	Tissue	Immunohistochemistry	TGF-β1expression was a significantly low-risk variable for death after pancreatectomy.	Odds ratio (95% CI): 0.441 (0.227–0.856) p = 0.0155.	Japan	Hashimoto et al. 2001[56]
	Plasma	ELISA	↓ levels in chemotherapy responders than non-responders	p = 0.032	Italy	Vizio et al. 2012 [65]
**VEGF**	Serum	ELISA	↓ levels in PDAC patients after radical resection	p = 0.003	Greece	Karayiannakis et al. 2003 [86]
	Serum	ELISA	↓ levels in PDAC patients after 1 cycle of gemcitabine and cisplatin combination chemotherapy.	p < 0.05	Korea	Bang et al. 2006 [73]

CI, confidence interval; BEV+CAPE+ RT, bevacizumab+capecitabine+ radiotherapy; ELISA, enzyme-linked immunosorbent assay; GEM, gemcitabine; IL, interleukin; NS, non-significant; OR, odds ratio; PDAC, pancreatic ductal adenocarcinoma; TGF-β1, transforming growth factor-beta 1; VEGF, vascular endothelial growth factor

#### Interleukin (IL)-1β

Studies that investigated IL-1β reported increased levels in PDAC patients [28, 29, 50, 53, 57, 59] with the exception of three studies: one identified lower serum concentrations [33], and two studies [60, 61] reported non-significant differences between patients and controls (Table 2). While IL-1β levels were lower in serum samples of PDAC patients compared to controls according to Bellone et al. [33], the same study reported increased gene expression levels in tissue specimen and strong staining by immunohistochemistry in cases. In three studies, increased levels of IL-1β were associated with poor prognosis: metastasis [28], shorter survival [33], and when analysed together with IL-6, was associated with poor overall survival and tumor progression [29] (Table 3).

#### IL-2

Poch et al. [57] reported lower serum levels of IL-2, whereas other studies [53, 62] detected higher levels in PDAC patients when compared to healthy controls. On the other hand, Ishikawa et al. [38] noted that IL-2 levels increased in PDAC patients after adoptive T-cell therapy, although it is not clear whether or not these alterations were significantly different between responders and non-responders. Certainly the study did not find any association between changes in IL-2 levels after adoptive T-cell therapy and patient overall survival (Table 4).

#### IL-6

The studies that investigated IL-6 reported increased levels in PDAC patients compared to healthy controls and individuals with chronic pancreatitis irrespective of the sample types and methods of measurement used [33, 36, 41, 43, 44, 49, 50, 53, 55, 58, 60, 63–68], with the exception of one study [69] that found non-significant differences between PDAC cases and controls (Table 2). When patients were stratified according to disease stage, Bellone et al. [33] found higher levels of IL-6 in patients with metastatic tumors (Union for International Cancer Control [UICC] stage IV) compared to those with locally extended tumors (UICC stages II and III) (Table 3). Furthermore, the prognostic potential of IL-6 was observed in other studies [29, 36, 44, 55, 58, 68, 70] but not in a study by Vizio et al. [65] (Table 3). Instead Vizio’s group [65] demonstrated that after gemcitabine and combination therapy (bevacizumab+capecitabine+radiotherapy), IL-6 levels decreased in PDAC patients but with no significant difference between responders and non-responders (Table 4).

#### IL-8

Many studies that investigated IL-8 reported increased levels in PDAC patients compared to healthy controls [25, 33, 34, 41, 49, 50, 53, 68, 71] (Table 2). In contrast, Zhang et al. [53] reported lower IL-8 levels in PDAC patients compared to participants with benign pancreatic diseases, similar to findings reported by Sakamoto et al. [19]. However, this study reported higher levels of IL-8 in patients with metastatic disease as opposed to participants without evidence of metastasis (Table 3). Further supporting the prognostic value of IL-8, Blogowski et al. [49] observed increased levels in PDAC patients with locally advanced and metastatic disease, while in a study by Dima et al [61] lower IL-8 levels of 9.27 pg/mL were found in patients who survived longer.

#### IL-10

One study reported lower levels of IL-10 in PDAC patients compared to healthy controls [62], while in other studies cases were characterized by higher levels [33, 49, 53, 57, 62, 66, 68, 72–74]. Moreover, increased IL-10 levels were associated with advanced cancer as reported by Wenger et al. [75] and Bellone et al. [33] (Table 2). Although not statistically significant, a response to therapy after 28 days lowered the concentration levels of IL-10 as noted by Bang et al. [73] (Table 4). On the other hand, a statistically significant decrease of IL-10 after combined chemotherapy was observed in another study conducted by Bellone et al. [72].

#### Transforming growth factor (TGF)

There was no agreement among studies that investigated TGF levels between PDAC patients and controls (Table 2). Breitbart et al. [66] reported decreased levels of TGF-β in serum sample of PDAC patients, whereas other studies reported increased levels in serum [33, 57, 74], plasma [65], pancreatic fluid [41] and tissue samples [22, 33]. On the other hand, Chuang et al. [27] found lower levels of TGF-α in urine samples of PDAC patients. Increased levels of TGF-β1 were associated with increasing risk of death from pancreatic cancer [40], shorter overall survival [65] and advanced disease stage in some studies [22, 33, 57], while in others studies a lower concentration was correlated with longer patient survival [33] (Table 3) and response to therapy [33, 65] (Table 4). In contrast, Hashimoto et al. [56] demonstrated a correlation between TGF-β expression in tumor samples and lower risk of PDAC and longer patient survival (Table 3). Sears et al. [76] and Culhaci et al. [77] found statistically non-significant association between TGF-β expression and PDAC.

#### Tumor necrosis factor alpha (TNF-α)

Four studies reported increased levels of TNF-α in PDAC patients compared to healthy controls [49, 53, 57, 61] (Table 2). However, when PDAC patients were compared to individuals with chronic pancreatitis, Zhang et al. [53] observed lower TNF-α levels in the former group. On the other hand, Wenger et al. [75] and Gabitass et al. [78] observed non-significant differences in levels of plasma TNF-α when comparing PDAC patients with healthy controls. Furthermore, findings from other studies [61, 75, 79] were suggestive of TNF-α having a prognostic role as increased levels were found in PDAC patients with metastasis, larger tumors and shorter survival (Table 3).

#### Microphage inhibitory cytokine-1 (MIC-1)

Increased serum levels of MIC-1 in PDAC patients compared to those with other pancreatic neoplasms, chronic pancreatitis, and healthy controls were reported in three studies [47, 52, 80] (Table 2). On the other hand, Baine et al. [37] found lower MIC-1 mRNA levels in PBMCs of patients. However, when PDAC patients were analysed separately, MIC-1 levels seemed to increase with tumor progression [37] (Table 3).

#### Macrophage colony-stimulating factor (M-CSF)

Increased M-CSF levels were found in PDAC patients than healthy controls [45, 46, 48, 81] (Table 2), and correlated with advanced PDAC stage [46, 48, 81] (Table 3).

#### Vascular endothelial growth factor (VEGF)

The majority of studies that examined VEGF profile in relation to PDAC reported elevated circulating levels and positive expression in tumor tissues of PDAC patients compared to those found in their normal counterparts [31, 32, 73, 82–84] (Table 2). However, when Sakamoto et al. [19] compared PDAC patients to individuals with pancreatitis and benign hepatobiliary diseases, lower levels were observed in the former group. On the other hand, Gabitass et al. in 2011 [78] reported statistically non-significant levels between PDAC patients and healthy controls, but increased levels when cases were compared to individuals with esophagus and gastric cancers. Vizio et al. [82] measured VEGF isoforms separately, detecting higher VEGF-A and lower VEGF-D levels in PDAC patients. In addition to elevated levels of VEGF, Chang et al. [84] found increased concentrations of the soluble VEGF receptor-1 (sVEGFR-1) in PDAC patients compared to healthy controls. The potential prognostic role of VEGF was noted in a few studies, demonstrating a correlation between increased levels and advanced PDAC stage (metastasis) and shorter survival [30, 31, 82, 83, 85, 86] (Table 3). For example, high levels of VEGF were associated with a favorable prognosis (hazard ratio; 95% confidence interval: 0.24; 0.09–0.57) as reported by Rahbari et al. [83]. On the other hand, higher VEGF/sVEGFR-1 ratio but not VEGF alone was association with poor prognosis in PDAC patients (HR 95% CI: 1.032; 1.007–1.056) according to Chang et al [84]. Findings from other studies were suggestive of a predictive role of VEGF in PDAC. For example, changes in serum VEGF levels were associated with gemcitabine and cisplatin combination chemotherapy in PDAC patients as reported by Bang et al. [73]. Vizio et al. [82] observed that VEGF levels decreased in PDAC patients after single (gemcitabine) or combination chemotherapy (gemcitabine combined with either oxaliplatin or 5-fluorouracil) (Table 4), but with no significant differences between responders and non-responders. Karayiannakis et al. [86] investigated serum levels of VEGF in PDAC patients compared to controls and found higher concentrations in cases, which decreased significantly after radical surgery.

### Diagnostic performance

Few cytokines were investigated for diagnostic performance in discriminating PDAC patients from those with other pancreatic diseases and/or healthy individuals (Table 5), some of which were compared to CA19-9 and CEA [45, 46, 52]. Some of these studies investigated panels of cytokines in comparison with CA19-9 [45, 46, 50, 51]. Individual cytokines exhibited poor diagnostic performance (sensitivity and specificity < 90%), except EGF (specificity = 100%), TGF-α (specificity = 100%), M-CSF (specificity = 95%), granulocyte-colony stimulating factor (G-CSF) (specificity = 95%), IL-23 (specificity = 94.9%), and macrophage migration inhibitory factor (MIF) (sensitivity and specificity = 100%). Cytokine panels showed superior diagnostic performance to CA19-9 alone. For example, the ‘IL-1β + IL-8 + CA 19–9’ panel had a sensitivity of 94.1% vs 85.9%, specificity of 100% vs 96.3%, and AUC of 0.984 vs 0.925 when compared to CA 19–9 alone in distinguishing PDAC patients from healthy controls [50]. For distinguishing PDAC patients from those with benign disease, a panel of IL-8, IL-6, IFN-gamma-inducible protein 10 (IP-10), PDGF and CA 19–9 when compared to CA 19–9 alone had an improved specificity (91.7% vs 66.7%) but at the expense of sensitivity (81.4% vs 88.4%).

**Table 5:** Diagnostic performance of cytokines investigated by selected studies.

Sensitivity		Specificity		AUC	Positive predictive value	Negative predictive value	Reference
**1 cytokine**	**Combination of cytokines**	**1 cytokine**	**Combination of cytokines**				
**PDAC vs HC:** EGF at 32.6 μg/g: creatinine = 13.3%; TGF-α at 18.8 μg/g creatinine = 0.0%	NA	**PDAC vs HC:** EGF at 32.6 μg/g: creatinine = 100%; TGF-α at 18.8 μg/g = 100%	NA	NA	NA	NA	Chuang et al. 1994 [27]
**PDAC vs non-cancer patients:** MIC-1at 1070 pg/ml = 71%; CA19–9 = 59%.	**PDAC vs non-cancer patients** MIC-1 + CA19-9 = 70%.	**PDAC vs non-cancer patients:** MIC-1 = 78%;CA19–9 = 88%.	**PDAC vs non-cancer patients:** MIC-1 + CA19-9 = 85%.	**PDAC vs noncancer:** MIC-1 = 0.81.	NA	NA	Koopmann et al. 2004 [51]
				**Periampullary adenocarcinomas vs noncancer:** MIC-1 = 0.79; CA19–9 = 0.77; MIC-1 + CA19–9 = 0.87.	NA	NA	
**PDAC vs HC:** SCF at 1285 ng/L = 98%; GM-CSF at 0.44 ng/L = 69%; M-CSF at 664 ng/L = 67%; G-CSF at 30.6 ng/L = 19%;IL-3 at 0.10 ng/L = 62%; CA-19-9 at 30x10^3^ U/L = 77%; CEA at 4.0 μg/L = 37%	NA	**PDAC vs HC:** SCF at 1285 ng/L = 17%; GM-CSF at 0.44 ng/L = 70%; M-CSF at 664 ng/L = 95%; G-CSF at 30.6 ng/L = 95%; IL-3 at 0.10 ng/L = 80%; CA-19-9 at 30x10^3^ U/L = 100%; CEA at 4.0 μg/L = 37%	NA	SCF = 0.9018; GM-CSF = 0.7703; M-CSF = 0.8461; G-CSF = 0.5133; IL-3 = 0.7141; CA 19–9 = 0.9146; CEA = 0.9091	SCF = 59%; M-CSF = 94%; CA 19–9 = 100%; CEA = 100%	SCF = 87%; M-CSF = 70%; CA 19–9 = 78%; CEA = 78%	Mroczko et al. 2005 [45]
**PDAC vs HC:** IL-8 at 23 pg/mL = 74%		**PDAC vs HC:**IL-8 at 23 pg/mL = 100%			NA	NA	Noh et al. 2006 [41]
**PDAC vs HC:** M-CSF = 37%; G-CSF = 26%; CA 19–9 = 74%; CEA = 40%	**PDAC vs HC:** M-CSF + GCSF = 52%; M-CSF + CA 19–9 = 84%; G-CSF + CA 19–9 = 81%	**PDAC vs HC:** M-CSF = 95%; G-SCF = 92%; CA 19–9 = 100%; CEA = 100%	**PDAC vs HC:** M-CSF + GCSF = 89%; M-CSF + CA 19–9 = 95%; G-CSF + CA 19–9 = 92%	M-CSF = 0.7191; G-CSF = 0.6576; CA 19–9 = 0.8886 CEA = 0.8720	NA	NA	Groblewska et al. 2007 [46]
**PDAC vs HC:** NR; **PDAC vs CP:** NR	NA	**PDAC vs HC:** NR; **PDAC vs CP:** NR	NA	**PDAC vs HC:** IL-6 = 0.9439; CA 19–9 = 0.8622; CEA = 0.8937.	NA	NA	Mroczko et al. 2010 [55]
				**PDAC vs CP;** IL-6 = 0.8433; CA 19–9 = 0.8097; CEA = 0.7390.	NA	NA	
**PDAC vs HC:** MIC-1 at 1.259 pg/mL: 81%; CA 19–9 at 34.3 U/mL: 81%.	NA	**PDAC vs HC:** MIC-1 at 1.259 pg/mL: 73% CA 19–9 at 34.3 U/mL: 97%.	NA	NA	NA	NA	Ӧzkan et al. 2011 [47]
**PDAC vs benign pancreatic diseases:** MIC-1 at 1.259 pg/mL: 62%; CA 19–9 at 34.3 U/mL: 81%.	NA	**PDAC vs benign pancreatic diseases:** MIC-1 at 1.259 pg/mL: 81%; CA 19–9 at 34.3 U/mL: 71%.	NA	NA	NA	NA	
**PDAC vs HC:** MIC-1 = 26.1%: CA 19–9 at > 37 U/mL = 74%; CA 19–9 at ≥ 61.7 U/mL = 70%	**PDAC vs HC:** MIC-1 + CA 19–9 + 3 proteins = 67%	**PDAC vs HC:** MIC-1 = 80%; CA 19–9 at > 37 U/mL = 27%; CA 19–9 at ≥ 61.7 U/mL = 80%	**PDAC vs HC:** MIC-1 + CA 19–9 + 3 proteins = 81%	**PDAC vs HC:** MIC-1 = 0.574; CA 19–9 = 0.719; MIC-1 + CA 19–9 + 3 proteins = 0.772	NA	NA	Baine et al. 2011 [37]
**PDAC vs CP:** MIC-1 = 37%; CA 19–9 at > 37 U/mL = 74%; CA 19–9 at ≥ 74.0 U/mL = 65%	**PDAC vs CP:** MIC-1 + CA 19–9 + 3 proteins = 67%	**PDAC vs CP:** MIC-1 = 80%; CA 19–9 at > 37 U/mL = 34%; CA 19–9 at ≥ 74.0 U/mL = 80%	**PDAC vs CP:** MIC-1 + CA 19–9 + 3 proteins = 83%	**PDAC vs CP:** MIC-1 = 0.640; CA 19–9 = 0.704 MIC-1 + CA 19–9 + 3 proteins = 0.820	NA	NA	
**PDAC vs HC:** M-CSF at 73 ng/L = 80%; SCF at 921 ng/L = 75.7%; IL-3 at 13 ng/L = 70%; CA 19–9 at 40 U/mL = 80%; CEA at 2.2 μg/L = 55%	**PDAC vs HC:** SCF + M-CSF = 97.5%	**PDAC vs HC:** M-CSF at 73 ng/L = 62.5%; SCF at 921 ng/L = 72.5%; IL-3 at 13 ng/L = 52.5%; CA 19–9 at 40 U/mL = 100%; CEA at 2.2 μg/L = 80%	**PDAC vs HC:** SCF + M-CSF = 46%	**PDAC vs HC:** M-CSF = 0.76; SCF = 0.70; IL-3 = 0.65; CA 19–9 = 0.91; CEA = 0.67	**PDAC vs HC:** SCF = 71.8%; M-CSF = 68.1%; IL-3 = 59.6%	**PDAC vs HC:** SCF = 76.3%; M-CSF = 75.8%; IL-3 = 63.6%.	Vasiliades et al. 2012 [48]
**PDAC vs HC:** MIC-1 ≥ 1.07 ng/mL = 90%; MIC-1 > 2.3 ng/mL = 62%; CA 19–9 at ≥ 37 U/mL = 83%; CA 19–9 at > 55 U/mL = 79%; **Stage 1/2 PDAC vs HC:** MIC-1 ≥ 1.07 ng/mL **=** 94%. MIC-1 > 2.3 = ng/mL = 81%. CA 19–9 at ≥ 37 U/mL = 71% CA 19–9 at > 54.1 U/mL = 74% **Stage 3/4 PDAC vs HC:** MIC-1 ≥ 1.07 ng/mL **=** 90%. MIC-1 > 2.3 ng/mL = 90%. CA 19–9 at ≥ 37 U/mL = 88%; CA 19–9 at > 54.1 U/mL = 83%.	NA	**PDAC vs HC:** MIC-1 ≥ 1.07 ng/mL = 46%; MIC-1 > 2.3 ng/mL = 63%; CA 19–9 at ≥ 37 U/mL = 67%; CA 19–9 at > 55 U/mL = 92%; **Stage 1/2 PDAC vs HC:** MIC-1 ≥ 1.07 ng/mL **=** 46%; MIC-1 > 2.2 = ng/mL = 64%.;CA 19–9 at ≥ 37 U/mL = 67%; CA 19–9 at > 54.1 U/mL = 92%; **Stage 3/4 PDAC vs HC:** MIC-1 ≥ 1.07 ng/mL **=** 46%; MIC-1 > 2.3 ng/mL = 58%; CA 19–9 at ≥ 37 U/mL = 67%; CA 19–9 at > 54.1 U/mL = 92%.	NA	**MIC-1 + CA19-9 (stage 1/2 PDAC vs HC):** from 0.8 to 0.82; **MIC-1 + CA19-9 (stage 3/4 PDAC vs HC):** from 0.89 to 0.94; **NGAL + MIC-1 + CA19-9 (stage 1/2 PDAC vs HC):** from 0.8 to 0.85; **NGAL + MIC-1 + CA19-9 (stage 3/4 PDAC vs HC):** from 0.89 to 0.94.	NA	NA	Kaur et al. 2013 [52]
**PDAC vs CP:** MIC-1 ≥ 1.07 ng/mL **=** 90%; MIC-1 > 2.3 ng/mL = 62%; CA 19–9 at ≥ 37 U/mL = 83%; CA 19–9 at > 62.2 U/mL = 79%; **Stage 1/2 PDAC vs CP:** MIC-1 ≥ 1.07 ng/mL **=** 94%; MIC-1 > 2.3 ng/mL = 76%.CA 19–9 at ≥ 37 U/mL = 71%; CA 19–9 at > 49.4 U/mL = 76%; **Stage 3/4 PDAC vs CP:** MIC-1 ≥ 1.07 ng/mL **=** 90%. MIC-1 > 2.3 ng/mL = 55%. CA 19–9 at ≥ 37 U/mL = 88%; CA 19–9 at > 186 U/mL = 70%.	NA	**PDAC vs CP:** MIC-1 ≥ 1.07 ng/mL **=** 30%; MIC-1 > 2.3 ng/mL = 62%; CA 19–9 at ≥ 37 U/mL = 78%; CA 19–9 at > 62.2 U/mL = 78%. **Stage 1/2 PDAC vs CP:** MIC-1 ≥ 1.07 ng/mL **=** 30%. MIC-1 > 2.3 ng/mL = 78%; CA 19–9 at ≥ 37 U/mL = 61%; CA 19–9 at > 49.4 U/mL = 74%; **Stage 3/4 PDAC vs CP:** MIC-1 ≥ 1.07 ng/mL **=** 30%;MIC-1 > 3.5 ng/mL = 91%;CA 19–9 at ≥ 37 U/mL = 61%;CA 19–9 at > 186 U/mL = 96%.	NA	**NGAL + MIC-1 + CA19-9 (stage 3/4 PDAC vs CP):** from 0.87 to 0.92; **MIC-1 + CA19-9 (stage 1/2 PDAC vs CP):** from 0.74 to 0.85; **MIC-1 + CA19-9 (stage 3/4 PDAC vs CP):** from 0.87 to 0.93; **NGAL + MIC-1 + CA19-9 (stage 1/2 PDAC vs CP):** from 0.74 to 0.86.	NA	NA	
**PDAC vs non-cancer:** IL-6 ≥ 4.92 pg/mL = 82.1%; IL-8 ≥ 51.15 pg/mL = 72.1%; IL-10 ≥ 7.35 pg/mL = 72.1%; IL-23 ≥ 32.5 pg/mL = 34.9% (exclusion); TNF-α ≥ 6.75 pg/mL = 76.7%; CA 19–9 = 74.4%	NA	**PDAC vs non-cancer:** IL-6 ≥ 4.92 pg/mL = 56.6%; IL-8 ≥ 51.15 pg/mL = 71.7%; IL-10 ≥ 7,35 pg/mL = 81.8%; IL-23 ≥ 32.5 pg/mL = 94.9% (exclusion); TNF-α ≥ 6.75 pg/mL = 60.6%; CA 19–9 = 80.8%	NA	**PDAC vs non-cancer:** IL-6 = 0.82; IL-8 = 0.71; IL-10 = 0.82; IL-23 = 0.65; TNF-α = 0.74	IL-6 = 46.3%; IL-8 = 52.5%; IL-10 = 63.3%; IL-23 = 75.0%; TNF-α = 45.8%; CA 19–9 = 62.7%.	IL-6 = 90.3%; IL-8 = 85.5%; IL-10 = 87.1%; IL-23 = 77.0%; TNF-α = 85.7%; CA 19–9 = 87.9%.	Blogowski et al. 2014 [49]
**PDAC vs HC:** CA 19–9 = 85.9%.	**PDAC vs HC:** IL-1β + IL-8 + CA 19–9 = 94.1%.	**PDAC vs HC:** CA 19–9 = 96.3%.	**PDAC vs HC:** IL-1β + IL-8 + CA 19–9 = 100%.	**PDAC vs healthy subjects:** In the training dataset: IL-8 + IL-1β + CA19-9 = 0.984 vs 0.925 (CA19-9 alone); In the test set: IL-8 + IL-1β + CA19-9 = 0.997 vs 0.975 (CA19-9 alone).	NA	NA	Shaw et al. 2014 [50]
**PDAC vs benign disease:** CA 19–9 = 53.6%.	**PDAC vs benign disease:** IL-1β + IL-8 + CA 19–9 **=** 92.9%.	**PDAC vs benign:** CA 19–9 = 84.4%.	**PDAC vs benign disease:** IL-1β + IL-8 + CA 19–9 = 57.8%.	**PDAC vs benign disease:** In the training dataset: IL-8 + IP-10 + IL-6 + PDGF + CA19-9 = 0.838 vs 0.678 (CA19-9 alone). In the test set: IL-8 + IP-10 + IL-6 + PDGF + CA19-9 = 0.884 vs 0.798 (CA19-9 alone).	NA	NA	
	**PDAC + obstructive Jaundice vs benign disease + obstructive jaundice:** IP-10 + IL-8 + IL-1β + PDGF = 74.5%.		**PDAC + obstructive jaundice benign disease + obstructive jaundice:** IP-10 + IL-8 + IL-1β + PDGF = na.	**PDAC with obstructive jaundice vs patients with benign disease and obstructive jaundice:** In the training dataset: IP-10 + IL-8 + IL-1β + PDGF = 0.810 vs 0.614 (CA19-9 alone);In the test set: IP-10 + IL-8 + IL-1β + PDGF = 0.857 vs 0.659 (CA19-9 alone).	NA	NA	
**PDAC vs CP:** CA 19–9 = 53.6%.	**PDAC vs CP:** IL-1β + IL-8 + CA 19–9 **=** 75.0%.	**PDAC vs CP:** CA 19–9 = 96.9%.	**PDAC vs CP:** IL-1β + IL-8 + CA 19–9 = 90.6%.	**PDAC vs CP:** In the training dataset: IL-8 + IL-6 + IP-10 + CA19-9 = 0.880 vs 0.758 (CA19-9 alone). In the test set: IL-8 + IL-6 + IP-10 + CA19-9 = 0.912 vs 0.848 (CA19-9 alone).	NA	NA	

CA 19–9, carbohydrate antigen 19–9; CEA, carcinoembryonic antigen; CP, chronic pancreatitis; HC, healthy controls; IL, interleukin; NA, not applicable; NGAL, neutrophil gelatinase-associated lipocalin; PDAC, pancreatic ductal adenocarcinoma; EGF, epidermal growth factor; G-CSF, granulocyte-colony stimulating factor; GM-CSF, granulocyte-macrophage colony-stimulating factor; IL, interleukin; IL-1F1, IP-10, IFN-gamma-inducible protein 10; M-CSF, macrophage colony-stimulating factor; MIC-1, macrophage inhibitory cytokine-1; NR, not reported; PDGF, platelet-derived growth factor; SCF, stem cell factor; TGF, transforming growth factor; TNF, tumor necrosis factors.

## Discussion

Cytokines play an important role as effector molecules in alerting and initiating immunological responses against pathogens and cancer cells. Alterations in their function may result in chronic disease progression through auto-inflammatory and auto-immune pathways [87, 88]. Cytokines have been the subject of extensive research for many years in relation to pathological disorders, and therefore, been considered biomarkers of disease states as well as treatment effectiveness. This review investigated the role of cytokines as diagnostic, prognostic and/or predictive biomarkers of PDAC, and has demonstrated incongruent results.

### The role of cytokines in PDAC diagnosis, prognosis, and prediction of treatment response

Six cytokines (IL-1β; IL-6, IL-8, VEGF, TGF, IL-10) have been consistently reported to be positively associated with PDAC, irrespective of biological material (serum, plasma, tissue, or peripheral blood mononuclear cells), method of measurement, and statistical analysis model used (Table 2). Specifically, IL-6, IL-8, VEGF, TGF, IL-10 were not only differentially expressed between PDAC and healthy controls but also between PDAC and pancreatitis patients [19, 41, 50, 53, 61, 70, 71, 74]. Only three studies investigated the potential diagnostic value of IL-1β; IL-6, IL-8, IL-10 for discrimination of PDAC patients from those with other pancreatic malignancies and diseases [49, 50, 55]. The performance characteristics of these individual cytokines were similar to that of CA19-9 in distinguishing PDAC patients from those with other pancreatic tumors and diseases as demonstrated by Blogowski et al. [49]. On the other hand, IL-6 performed better than CA19-9 and CEA when used to discriminate PDAC patients from healthy and chronic pancreatitis individuals [53]. Shaw et al. [50] demonstrated that IL-1β, IL-6 and IL-8 improved the diagnostic performance of CA19-9 for discriminating PDAC from benign pancreatic diseases, jaundice, and chronic pancreatitis when they were in distinct panels with CA19-9, IP-10, and PDGF. On their own, these studies are insufficient to support or rule-out the use of IL-1β, IL-6 and IL-8 as biomarkers for PDAC diagnosis, either individually or as part of distinct panels of cytokines. It should be noted that the diagnostic performance of these cytokines was conducted using one set of participants, and not tested in a validation test sample. Ideally, a diagnostic biomarker should be non-invasive with close to 100% specificity and sensitivity to the target disease. Moreover, with regard to PDAC diagnosis, the biomarker should be able to distinguish affected patients from those with other pancreatic diseases, and this be tested successfully in a different study sample. Non-invasive laboratory tests that are currently used such as CA 19–9, those for liver function, and PAM4 monoclonal antibody are non-specific as they can also be expressed in other pancreatic diseases [89–91]. This poses challenges for accurate PDAC diagnosis because the majority of patients who get a confirmed diagnosis of PDAC by imaging and pathological examination initially present with moderate to severe pancreatitis [92]. A non-invasive PDAC biomarker is required that can distinguish affected patients from those with similar symptoms at presentation without undergoing imaging and/or biopsy examination.

Furthermore, all six inflammatory cytokines were reported to have a potential prognostic value, with higher levels associated with advanced PDAC stage (metastasis) and poor patient survival [19, 22, 28–31, 33, 36, 40, 44, 49, 55, 57, 58, 68, 70, 75, 82, 83, 85, 86]. The role of inflammation in PDAC initiation and progression has been well demonstrated in animal models. Particularly in mouse models, experimentally induced chronic pancreatitis combined with targeted expression of oncogenic mutant *Kras*^*G12V*^ in pancreatic acini have been shown to induce PDAC formation in mouse models [93]. In genetically modified mouse models, inflammation has been shown to promote epithelial-mesenchymal transition and invasiveness [94]. Furthermore, Steele et al [95] observed that C-reactive protein, a marker of systemic inflammation, was associated with PDAC recurrence. Thus, cytokines such as IL-6, IL-1α, and TNF-α have been identified as excellent therapeutic targets. This is further supported by evidence showing that levels of cytokines such as IL-2, IL-6, VEGF, and TGF were altered upon introduction of adoptive T-cell therapy or chemotherapy or by radical surgery but showed no significant association with patient response to treatment [38, 56, 65, 72, 73, 86]. Similarly, Ishikawa et al. [38] found elevated IL-10 levels in PDAC patients after adoptive T-cell therapy with no statistical significance. Further studies are warranted to determine whether or not the changes in cytokine levels in response to treatment regime render them potential predictive biomarkers by conducting statistical tests of association between the above-mentioned cytokines and treatment response. Notwithstanding, anti-TNF therapy has been demonstrated in orthotopic PDAC models to reduced primary tumor size and metastases [96]. However, this data has not yet been translated to clinical trial. IL-6 monoclonal antibodies, Siltuximab and Tocilizumab, which bind to the soluble form of the IL-6 receptor are available for trial and currently under assessment in ovarian cancer [97]. Whether all or some of these cytokines have clinical diagnostic, prognostic or predictive biomarker value requires further investigation.

### Analysis of potential reasons for heterogeneity observed among studies

In contrast to the above-mentioned observations, several studies reported either lower levels or non-significant differences in IL-1β; IL-8 and VEGF concentration between the study groups [19, 33, 41, 50, 53, 60–62, 71, 78]. Inconsistent findings were also noted for other cytokines. Heterogeneity in study results may be attributed to flaws and/or variation in study design and execution. Twenty-nine diagnostic studies compared PDAC patients with healthy individuals, and one of these studies reported colonoscopy examination of the control group [51]. The challenge with using healthy control is that they often do not undergo imaging examination to rule out any asymptomatic pancreatic abnormalities. If a case-control design is used, it is recommended to compare the patient group with multiple control groups such as individuals with other pancreatic diseases with similar presenting symptoms, and/or different cancer types for accurate estimation of specificity [98].

As the biomarker development proceeds to qualification stages, studies focus on confirming the association between a marker and disease to determine its sensitivity over specificity. Following this stage is the verification and validation of the biomarkers in a broader selection of cases and controls to account for biological and environmental variations in a population for which the biomarker is intended. This stage determines the ability of a biomarker to specifically identify true positives and negatives. A well-designed diagnostic accuracy test is conducted in a consecutively or randomly selected series of patients with a suspected target condition; in which sensitivity, specificity, positive and negative predictive values, diagnostic odds ratio and receiver operating characteristic (ROC) curves are analysed. Eleven of 44 diagnostic studies in the current systematic review conducted an accuracy test, but not to the full extent: eight of the 11 studies [37, 44–46, 50–53] determined sensitivity, specificity, and ROC tests. The selection of study population, particularly by studies that conducted diagnostic accuracy tests, was not as recommended for the specific type of studies rather used a case-control design. The use of only case-control group in the validation stage may overestimate the sensitivity and specificity of the biomarker being investigated [99, 100].

The assessment of prognostic studies is relatively new and not as well developed as diagnostic studies. However, studies should clearly establish prognostic value of a biomarker by demonstrating a significant association between the biomarker and outcome, independent of treatment. Apart from duration of follow-up, which varied and ranged from 8 weeks to 3 years, other methodological parameters that cause heterogeneity in study results were generally similar to those observed among diagnostic biomarker studies. From this point onwards, these parameters will be discussed encompassing both diagnostic and prognostic studies. Variations in the spectrum of study populations introduced another source of heterogeneity in study results. There was poor reporting on the demography and lifestyle factors (patient spectrum) of the study population in most studies. Only three studies reported on ethnicity of the study population [37, 40, 66]. The performance of biomarkers varies according to ethnicity, and its clinical validity is limited to the test population due to associated biological and environmental factors in specific populations. It is therefore important that studies clearly define the population of interest [16]. This is specifically relevant in the context of this review as genetic variations of cytokine genes among different population groups may have variable effects on the translation and concentration of their respective proteins and subsequently on the presentation of disease phenotypes [101–103]. This too may explain some of the inconsistent findings between studies. It is also noteworthy that cytokines are redundant in their functions [104], and that PDAC itself is thought to occur as a consequence of interactions between multiple genetic defects and various environmental factors [105]. Furthermore, it is recommended that participants be matched according to lifestyle factors such as alcohol consumption, smoking, physical activity, body mass index, and other related health conditions that may influence alteration of a specific biomarker under investigation [106]. Most of these factors were not reported by the studies in the current systematic review.

Studies were assessed for methodological variations that affect the adequacy and accuracy of cytokine measurement. Parameters such as sample procurement timing, sample handling and storage, and the choice of plasma or serum collected in different blood collection tubes types may affect adequate and accurate measurements of cytokine levels in biological samples [107,108–111]. There was poor reporting on these experimental parameters, by some [25, 27, 28, 30, 31, 33, 43, 45, 51, 59, 60, 62, 67, 69, 71, 73, 77, 81] but not all studies. Furthermore, the use of different sample types and detection methods added another confounding factor. For example, in a study by Bellone et al. [33] varying concentration levels of IL-1β were detected on serum and tissue samples of patients. Similar observations were noted for other cytokines in different studies [19, 23, 37, 47, 83], variations that may be explained by different detection platforms (ELISA vs gene array and radio-immunoassay) in addition to sample types used (serum vs tissue). Not all studies in the current review reported on sample handling and storage, thus limiting our analysis and evaluation of the quality of methodologies used.

A limitation of this review is that only one, albeit large, biomedical research database was used. However, the search was supplemented with records identified in reference lists of review manuscripts and meta-analyses. Furthermore, citations were limited to the English language.

In summary, our review highlights the paucity of evidence in relation to cytokines that may be used to develop diagnostic, prognostic and treatment prediction strategies for PDAC. The concentrations of six cytokines (IL-1β; IL-6, IL-8, VEGF, TGF, IL-10) were consistently reported to be increased in PDAC patients irrespective of sample type, method of measurement, and statistical model used. However, these cytokines have not been tested for their diagnostic performance by many studies, and are yet to be validated in different study population sets. Diagnostic performance tests should be conducted in light of the emerging evidence suggesting that their ability to discriminate PDAC from non-malignant pancreatic diseases and healthy controls improves when they are tested as a panel as demonstrated by Shaw et al [50]. Interleukin-1β, -6, -8, -10, VEGF, and TGF were associated with the severity of PDAC (i.e., metastasis, tumor size, and advanced stage), suggestive of a role as prognostic biomarkers. Clinical evaluation of these findings is required to demonstrate the association of the above-mentioned cytokines with PDAC outcome (severity and/or patient survival) independent of therapeutic intervention, and its effect on cytokines. Of these six cytokines, four (IL-6, IL-10, VEGF, TGF) together with IL-2 were altered after patients received treatment (surgery or chemotherapy). However, it should be noted that based on these findings the alterations observed were not significantly different between patients who responded to therapy and non-responders, and no statistical tests of association between the above-mentioned cytokines and treatment response were conducted. Further studies are therefore required to evaluate the clinical value of cytokines as diagnostic, prognostic, or predictive biomarkers. These studies should be conducted prospectively with well-defined homogeneous populations that should be followed-up for regular, specified time periods. Furthermore, consensus guidelines addressing cytokine laboratory methodology for measurements are needed to conduct reliable studies that may accurately identify diagnostic, prognostic and/or predictive biomarkers for PDAC.

## Supporting Information

S1 File PRISMA checklist. PRISMA for Abstracts Checklist. (PDF)

S1 Table MEDLINE search strategy (from inception to July 2015). (DOCX)

S2 Table Description of studies included in the systematic review. (DOCX)

S3 Table (a) and (b). Study characteristics and quality assessment of included diagnostic studies.
(XLSX)
