# Supplementary material for: Cytokines as Biomarkers of Pancreatic Ductal Adenocarcinoma: A Systematic Review
Source: PLoS One. 2016 May 12;11(5):e0154016. doi: 10.1371/journal.pone.0154016 (PMC4865360; doi:10.1371/journal.pone.0154016)
Supplement: S1 File — (PDF) [file pone.0154016.s001.pdf]

PRISMA 2009 CHECKLIST				
**Number**	**Section**	**Checklist item**	**Check if complete**	**Reference page number**
	**TITLE**			
1	Title.	Identify the report as a systematic review or meta-analysis or both.	√	1
	**ABSTRACT**			
2	Structured summary.	Provide a structured summary to include, as applicable: objectives; methods (data sources, study eligibility criteria, synthesis methods); results (key findings); conclusions (implications of key findings).	√	2
	**BACKGROUND**			
3	Rationale.	Describe the rationale for the review in the context of what is already known.	√	3-4
4	Objective.	The research question.	√	4
	**METHODS**			
5	Eligibility criteria.	Characteristics used as criteria for inclusion.	√	4
6	Information sources.	Key databases searched.	√	4
7	Search strategy.	Full electronic search strategy, including any limits.	√	S1 Table
8	Data extraction.	List and define all variables for which data were sought.	√	5
9	Risk of bias.	Describe methods used for assessing risk of bias of individual studies.	√	5-6

	**RESULTS**			
10	Study selection.	Number and type of included studies and participants and relevant characteristics of studies.	√	6 and Figue 1
11	Study characteristics.	For each study, present characteristics for which data were extracted and provide citation.	√	6-8 and S2 Table
12	Quality assessment of included studies (risk of bias).	Present data on risk of bias of each study and, if available, any outcome level assessment.	√	8-9 and Table 1 (page 33)
13	Results on cytokine profile and its relevance to the outcome/disease.	For each study, present characteristics for which data were extracted (e.g., results on statistical analyses).	√	10-15 and Tables 2-5 (pages 34-57)
	**DISUCSSION**			
14	Interpretation.	General interpretation of the results and important implications.	√	15-22
15	Strengths and limitations of evidence.	Brief summary of strengths and limitations of evidence.	√	22
16	Conclusion		√	22
	**FUNDING**			
17	Primary source of funding for the review.	Primary source of funding for the review.	No funding	Not applicable

Reference: Moher D, Liberati A, Tetzlaff J, Altman DG, The PRISMA Group (2009). Preferred Reporting Items for Systematic Reviews and Meta-Analyses: The PRISMA Statement. PLoS Med 6(6): e1000097.
