# Supplementary material for: Cytokines as Biomarkers of Pancreatic Ductal Adenocarcinoma: A Systematic Review
Source: PLoS One. 2016 May 12;11(5):e0154016. doi: 10.1371/journal.pone.0154016 (PMC4865360; doi:10.1371/journal.pone.0154016)
Supplement: S1 Table — (DOCX) [file pone.0154016.s002.docx]

## Table S1. MEDLINE search strategy (from inception to July 2015).

| **Search** | **Query** | **Number of hits** |
| --- | --- | --- |
| #1 | Search "pancreatic cancer" | [23876](http://www.ncbi.nlm.nih.gov/pubmed/?cmd=HistorySearch&querykey=1) |
| #2 | Search ((((cytokines) OR "Proinflammatory cytokines") OR "Anti-inflammatory cytokines") OR "Chemotactic cytokines") OR "angiogenic cytokines" | [605435](http://www.ncbi.nlm.nih.gov/pubmed/?cmd=HistorySearch&querykey=12) |
| #3 | Search ((((((((((((((((((((((((((((((((interleukin) OR interleukin 2") OR IL-2) OR "interleukin 3") OR IL-3) OR "interleukin 4") OR IL-4) OR "interleukin 5") OR IL-5) OR "interleukin 6") OR "interleukin 9") OR IL-9) OR "interleukin 10") OR IL-10) OR "interleukin 13") OR IL-13) OR "interleukin 17E) OR IL-17E) OR "interleukin 25") OR IL-25) OR "interleukin 33") OR IL-33) OR "interleukin 22") OR IL-22) OR "interleukin 23") OR IL-23) OR "interleukin 12") OR IL-12) OR "interleukin 1 alpha") OR "interleukin 1 beta") OR "interleukin 15") OR IL-15) OR "interleukin 1 receptor antagonist | [46270](http://www.ncbi.nlm.nih.gov/pubmed/?cmd=HistorySearch&querykey=2) |
| #4 | Search ("interferon gamma") OR INFg | [76111](http://www.ncbi.nlm.nih.gov/pubmed/?cmd=HistorySearch&querykey=3) |
| #5 | Search ((("Tumor necrosis factor alpha") OR TNFa) OR "Tumor necrosis factor beta") OR TNFb | [153006](http://www.ncbi.nlm.nih.gov/pubmed/?cmd=HistorySearch&querykey=4) |
| #6 | Search (("Monocyte chemoattractant protein-1") OR CCL2) OR MCP-1 | [19289](http://www.ncbi.nlm.nih.gov/pubmed/?cmd=HistorySearch&querykey=5) |
| #7 | Search ("interferon-inducible protein-10") OR IP-10 | [3137](http://www.ncbi.nlm.nih.gov/pubmed/?cmd=HistorySearch&querykey=6) |
| #8 | Search ("Transforming growth factor alpha") OR TGFa | [5663](http://www.ncbi.nlm.nih.gov/pubmed/?cmd=HistorySearch&querykey=7) |
| #9 | Search ("vascular endothelial growth factor") OR VEGF | [64907](http://www.ncbi.nlm.nih.gov/pubmed/?cmd=HistorySearch&querykey=8) |
| #10 | Search ("interleukin 8") OR IL-8 | [31938](http://www.ncbi.nlm.nih.gov/pubmed/?cmd=HistorySearch&querykey=9) |
| #11 | Search ("Epidermal growth factor") OR EGF | [70940](http://www.ncbi.nlm.nih.gov/pubmed/?cmd=HistorySearch&querykey=10) |
| #12 | Search animals | [5503489](http://www.ncbi.nlm.nih.gov/pubmed/?cmd=HistorySearch&querykey=13) |
| #13 | ("pancreatic cancer") AND ((((((((((((((((((((((((cytokines) OR "Proinflammatory cytokines") OR "Anti-inflammatory cytokines") OR "Chemotactic cytokines") OR "proangiogenic cytokines")) OR (("interleukin 2") OR IL-2)) OR (("interleukin 3") OR IL-3)) OR (("interleukin 4") OR IL-4)) OR (("interleukin 5") OR IL-5)) OR (("interleukin 6") OR IL-6)) OR (("interleukin 9") OR IL-9)) OR (("interleukin 10") OR IL-10)) OR (("interleukin 13") OR IL-13)) OR ((("interleukin 17E") OR IL-17E) OR IL-25)) OR (("interleukin 33") OR IL-33)) OR (("interleukin 22") OR IL-22)) OR (("interleukin 23") OR IL-23)) OR (("interleukin 12") OR IL-12)) OR (("interleukin 1 alpha") OR IL-1a)) OR (("interleukin 1 beta") OR IL-1b)) OR (("interleukin 15") OR IL-15)) OR (("interleukin 1 receptor antagonist) OR IL-1RA)) OR ((" AND interferon ganmma AND ") OR INFg)) OR ((((" AND Tumor necrosis factor alpha AND ") OR TNFa) OR " AND Tumor necrosis factor beta AND ") OR TNFb)) OR (((" AND Monocyte chemoattractant protein-1 AND ") OR CCL2) OR MCP-1)) OR ((" AND interferon-inducible protein-10 AND ") OR IP-10)) OR ((" AND Transforming growth factor alpha AND ") OR TGFa)) OR ((" AND vascular endothelial growth factor AND ") OR VEGF)) OR ((" AND interleukin 8 AND ") OR IL-8)) OR ((" AND Epidermal growth factor OR EGF)))) NOT animals | 1071 |
