# Supplementary material for: Cytokines as Biomarkers of Pancreatic Ductal Adenocarcinoma: A Systematic Review
Source: PLoS One. 2016 May 12;11(5):e0154016. doi: 10.1371/journal.pone.0154016 (PMC4865360; doi:10.1371/journal.pone.0154016)
Supplement: S2 Table — (DOCX) [file pone.0154016.s003.docx]

**S2 Table. Description of studies included in the systematic review.**

| **Reference** | **Study design** | **Country(population)** | **Number of Cases** | **Number of Controls** | **% men** | **Age group** | **Sample type** | **Cytokine measured** | **Method of detection** | **Statistical analysis** |
| --- | --- | --- | --- | --- | --- | --- | --- | --- | --- | --- |
| Chuang et al. 1994 [[28](#_ENREF_28)] | Retrospective | China | 15 PDAC | 40 healthy controls | NR | 27 - 84 | Urine | EGF; TGFα | Radioimmunoassay | Receiver operation curve; likelihood ratio; specificity; sensitivity; diagnostic accuracy; Spearman correlation |
| Falconer et al. 1994 [[69](#_ENREF_69)] | Retrospective | United Kingdom (ethnicity not specified) | 21 pancreatic cancer (7 stage II; 8 stage III; 6 stage IV) | 16 patients admitted for minor elective surgical procedures without evidence of infection or inflammation | 67.6 | 52 - 59 | Serum | TNF; IL-6 | ELISA | Two-tailed Student's unpaired t test |
| Basso et al. 1995 [[29](#_ENREF_29)] | Retrospective | Italy (ethnicity not specified) | 30 PDAC (5 stage 1; 8 stage 2; 6 stage 3; 11 stage 4); | 19 healthy controls; 20 chronic pancreatitis; 13 type 1 diabetes; 14 liver cirhosis | 57.3 | 24 - 89 | Serum | IL-1α/IL-1F1; IL-1β/IL-1F2 | Radioimmunoassay | ANOVA one-way; linear regression; Spearman's correlation |
| Fogar et al. 1998 [[60](#_ENREF_60)] | Retrospective | Italy (ethnicity not specified) | 18 pancraetic cancer (5 liver metastases, omental, 4 mesenteric infiltration, local metastases); | 20 healthy controls; 22 chronic pancreatitits | 60 | 17 - 83 | Serum | IL-6; IL-1β | ELISA | Kruskal-Wallis; Mann-Whitney |
| Fujimoto et al. 1998 [[31](#_ENREF_31)] | Prospective (follow-up: 2.4 - 82 months) | Japan | 50 PDAC who had undergone resection (6 stage 1; 13 stage 2; 15 stage 3; 16 stage 4) | NR | 56 | 41 - 89 | Tissue | VEGF; PD-ECGF | Immunohistochemistry; western blotting; reverse transcriptase-polymerase chain reaction | Student's t test; Kaplan-Meier method; log rank test; generalised Wilcoxon test |
| Gansauge et al. 1998 * | Prospective (follow-up: 6 - 8 weeks) | Germany (ethnicity not specified) | 101 PDAC (16 stage 1, 60 stage II and III, 25 stage IV) | 43 healthy controls; ; 89 chronic pancreatitis; 16 pancreatic cystadenocarcinoma | 38.6 | NA | Serum | IL-2Rα | ELISA (Immunometric assay) | Student's t test; Kaplan-Meier method; Multiple regression analysis |
| Okada et al. 1998 [[63](#_ENREF_63)] | Retrospective | Japan | 55 histologically-proven pancreatic cancer (tubular adenocarcinom, adenosquamous and mucinous carcinoma) | 20 normal healthy with no evidence of infection or inflammation; 25 chronic pancreatitis | 69 | 53 - 69 | Serum | IL-6 | ELISA | Chi square |
| Ikeda et al. 1999 [[32](#_ENREF_32)] | Prospective (5 - 62 months) | Japan | 40 pancreatic cancer (6 with distant metastasis underwent surgery) receiving intraoperative radiation therapy | NR | 75 | 47 - 80 | Tissue | VEGF; PD-ECGF | Reserve transcriptase-PCR; immunohistochemistry | Chi square or Mann-Whitney test; Pearson's correlation; Kaplan-Meier method; Cox proportional hazard regression model |
| Wenger et al. 1999 [[74](#_ENREF_74)] | Retrospective | NA | 28 with pancreatic carcinoma | 20 healthy controls | 42.9 | 49-68 | Plasma | IL-6 | ELISA | NPAR test; Logistic regression analysis |
| Barber et al. 1999 [[108](#_ENREF_108)] | Retrospective | United Kingdom (ethnicity not specified) | 13 unresectable pancreatic cancer (advanced): 7 stage II; 1 stage III; 5 stage IV | 6 with no active medical condition | NA | 50 - 62 | Serum | IL-6; IL-6R; TNF-R | indirect ELISA | Mann-Whitney test; Spearman correlation |
| Plate et al. 1999 [[62](#_ENREF_62)] | Retrospective | Not specified | 26 (stage III and IV and on therapy, 20 donated blood a week after receiving treatment while 6 donated blood before chemotherapy). | 12 normal healthy donors | 42.31 | 24 - 84 | Serum and PBMC | IL-2, IFN-γ, IL-4, IL-10, TNF-α, IL-1β, IL-12 receptor | quantitative PCR (RT-PCR); ELISA | Chi-squared test; non-parametric Kruskal-Wallis and Mann-Whitney test; scatter plots and Spearman correlation |
| Itakura et al. 2000 [[33](#_ENREF_33)] | Retrospective | Not specified | 15 PDAC (10 grade 2 and 5 grade 3; 2 stage II, 10 stage III, 3 stage III) who underwent surgery | 9 normal healthy donors | 45.83 | 22 - 77 | Tissue | VEGF | Immunohistochemistry; Northern blot | Chi square |
| Barber et al. 2000 [[59](#_ENREF_59)] | Prospective | United Kingdom (ethnicity not specified) | 64 (unresectable pancreatic cancer, IL-1beta measured in 22 patients): 30 stage II, 8 stage III, 26 stage IV | 101 healthy controls | 62.4 | 36 - 72 | Serum | IL-1β | ELISA | Chi-square test; Spearman correlation; Mann-Whitney; Kaplan-Meier; log rank test |
| Hashimoto et al. 2001 [[54](#_ENREF_54)] | Retrospective | Japan | 62 PDAC underwent pancreatectomy; 34 received adjuvant chemotherapy after surgery | NA | 46.77 | 35 - 79 | Tissue | TGF-β | Immunohistochemistry | Chi-square test; Kaplan-Meier; Log-rank test; Cox proportional hazard model |
| Karayiannakis et al. 2001 [[76](#_ENREF_76)] | Retrospective | Not specified | 63 PDAC (19 stage I; 7 stage II; 33 stage III; 4 stage IV) not receiving therapy | NA | 39.68 | 64 - 70 | Serum | TNF-α | ELISA | Fischer's exact test |
| von Bernstorff et al. 2001 [[73](#_ENREF_73)] | Retrospective | Germany (ethnicity not specified) | 116 PDAC | 77 healthy controls; 31 acute pancreatitis; 39 benign illness | 54.4 | 18 - 81 | Serum | IL-10; TGF-β1/2 | ELISA | Variance analysis or Dunnett T3; chi-squared and Fisher's exact test |
| Nagakawa et al. 2002 [[83](#_ENREF_83)] | Prospective (3.6 - 136.6 months) | Japan | 32 with resected PDAC (18 with postoperative liver metastasis; 14 without): 3 in stage I; 2 in stage II; 13 in stage III; 14 in stage IV. | NA | 75 | 44 - 77 | Tissue | VEGF | Immunohistochemistry | Chi-square; Fischer's exact test; Student t-test |
| Yue et al. 2002 [[23](#_ENREF_23)] | Retrospective | China | 32 pancreatic cancer patients who received operation: 19 with high-differentiated and 13 with moderate or low-differentiated. 20 had lymph node metastasis | Adjacent pancreatic tissues from 32 patients used as controls | NA | NA | Tissue | TGF-β1 | Immunohistochemistry | Chi-squared test; Wilcoxon's rank-sum test |
| Karayiannakis et al. 2003 [[79](#_ENREF_79)] | Prospective (1 - 59 months) | Not specified | 58 PDAC: 46 with distant metastasis and 40 with lymph node metastasis. 13 stage I; 5 stage II; 28 stage III; 12 stage IV. 18 patients underwent radical resection and 40 with unresectable tomours underwent either pallitative bypass surgery or endoscopic stenting. | 51 healthy controls | 60.55 | 42 - 93 | Serum | VEGF | ELISA | Mann-Whitney U test; Wilcoxon rank test; Kruskal-Wallis; Kaplan-Meier; Cox proportional regression analysis; log-rank test |
| Ebrahimi et al. 2004 [[68](#_ENREF_68)] | Retrospective (followed for 16 months) | USA (ethnicity not specified) | 51 PDAC | 62 healthy controls | 58.8 | 43-79 | Serum | IL-1β, IL-1α, IL-1RA, IL-6, IL-8, IL-10, TNF-α, VEGF | ELISA | Mann-Whitney U test; Cox proportional regression analysis; log-rank test; Spearman rank correlation |
| Koopmann et al. 2004 [[51](#_ENREF_51)] | Retrospective | Australia (ethnicity not specified) | 80 PDAC | 97 healthy controls undergoing screening colonoscopy; 77 chronic pancreatitis | 24.42 of cases | 55 - 78 | Serum | MIC-1 | *In situ* hybridisation; immunohistochemistry; tissue | Receiver operation curve; Student's t test; linear regression model |
| Mroczko et al. 2004 [[78](#_ENREF_78)] | Retrospective | Poland | 47 PDAC (19 stage III, 20 stage IVA, 8 stage IVB) | 35 healthy controls; 27 chronic pancreatitis | 56.89 | 26 - 86 | Serum | SCF; M-CSF | ELISA | Mann-Whitney U-test; Spearmann rank correlation |
| Sears et al. 2004 [[109](#_ENREF_109)] | Retrospective (cases dated 1993 through 1999) | USA (ethnicity not specified) | 120 PDAC: 18 stage I; 24 stage II; 22 stage III; 54 stage IV. 22 received chemo/radiation; 20 received surgery; 82 no treatment. | NR | 55.8 | 57 - 79 | Biopsy obtained through fine-needle aspiration | TGF-β | Immunohistochemistry | Kaplan-Meier survival analysis; Cox proportional hazard regression; Spearman correlation; chi-square test |
| Culhaci et al. 2005 [[110](#_ENREF_110)] | Prospective | Turkey | 63 PDAC undergoing pancreatic surgery for primary cancer without chemo or radiation therapy. 4 stage I; 23 stage II; 3 stage III; 33 stage IV. 39 with lymph node metastasis; 44 perineural invasion; 33 distant metastasis. | NR | 42.9 | 42 - 82 | Tissue | TGF-β1 | Immunohistochemistry | Chi-square and Fishers exact test; Kendall's tau-b and Spearman correlation |
| Mroczko et al. 2005 [[45](#_ENREF_45)] | Retrospective | Poland | 48 PDAC (15 stage III, 23 stage IV) | 40 healthy controls; 23 chronic pancreatitis | 53.15 | 21 - 86 | Serum | SCF; IL-3; GM-CSF; G-CSF; M-CSF | ELISA | Mann-Whitney U-test; Spearman rank correlation |
| Martignoni et al. 2005 [37] | Retrospective | Germany | 41 PDAC (14 with cachexia) | 21 healthy controls; 8 chronic pancreatitis | NR | 58.8-78.5 | Serum and tissue | IL-6 | ELISA; Immunohistochemistry; |  |
| Bang et al. 2006 [[72](#_ENREF_72)] | Prospective (1-28 days) | Korea | 13 stage IV, distant metastatic pancreatic cancer undergoing gemcitabine chemotherapy and cisplatin treatment | 7 healthy controls | 65 | 47 - 69 | Serum | VEGF; IL-10 | ELISA | Mann-Whitney test; Kruskal-Wallis test |
| Bellone et al. 2006 [[34](#_ENREF_34)] | Prospective (6 - 29 months) | Italy (ethnicity not specified) | 65 PDAC: 24 advanced non-resectable PDAC receiving only supportive treatment | 30 healthy donors. 9 tumour-free pancreatic cancer tissues; 41 underwent surgical resection | 57 | 24 - 65 | Tissue and serum | pro-inflammatory: IL-1β; IL-2; IL-6; IL-8; IL-12p40; IL-18; IFN-γ. Anti-inflammatory: IL-10; IL-11; IL-13 and TGF-β | Immunohistochemistry and quantitative PCR | Fisher's exact or Chi-square test; Mann-Whitney or Kruskal-Wallis test; log-rank test; REST (for analysing mRNA expression profile) |
| Koopmann et al. 2006 [[111](#_ENREF_111)] | Retrospective | USA (ethnicity not specified) | 50 PDAC with resectable cancer | 50 age/sex-matched healthy controls; 50 chronic pancreatitis | 44 PDAC; 50 chronic pancreatitis; 27 controls | 42 - 78 | Serum | MIC-1 | ELISA | Logistic regression analysis; Receiver operation curve analysis |
| Lin et al. 2006 [[40](#_ENREF_40)] | Prospective (mean follow-up of 10 years) | Japan | 85 PDAC | 252 | 45.7 | 40 - 79 | Serum | TGF-β1 | ELISA | Logistic regression analysis |
| Noh et al. 2006 [[41](#_ENREF_41)] | Retrospective | USA (ethnicity not specified) | 38 pancreatic cancer | 41 with normal pancreas; 39 chronic pancreatitis | 44.92 | 24 - 88 | Pancreatic juice | IL-6; IL-8; TGF-β1 | 2-site chemiluminescent immunometric assay; ELISA | Multiple regression analysis with a forward stepwise selection; Receiver operation curve analysis |
| Poch et al. 2007 [[55](#_ENREF_55)] | Prospective | Not specified | 32 pancreatic cancer patients, 15 underwent resection and 17 palliative operation. 3 stage I; 2 stage II; 15 stage III; 12 stage IV. | 24 healthy controls | 62.5 | 48 - 83 | Serum | TNF-α; IL-1β; IL-2; IL-10; IL-12; IL-18; IL-1RA; sIL-2R; TGF-β1 | ELISA | Student *t* test |
| Groblewska et al. 2007 [[46](#_ENREF_46)] | Retrospective | Poland | 62 pancreatic cancer, 17 underwent surgery. Classification: 8 stage II; 14 stage III; 17 stage Iva; 23 stage IVb. | 65 healthy controls | 59.8 | 21 - 88 | Serum | G-CSF; M-CSF | ELISA | Mann-Whitney U-test; Kruskal-Wallis test; Spearmann correlation; Log-rank test (univariate analysis of survival); Cox proportional hazard model (multivariate forward stepwise analysis). |
| Frick et al. 2008 [[35](#_ENREF_35)] | Retrospective | Germany (ethnicity not specified) | 15 pancreatic cancer (10 with lymph node metastasis); 6 cancer of the papilla | 17 patients with pancreatic cystadenoma; 12 with chronic pancreatitis | 63.16 | 32 - 79 | Tissue | IL-8 |  | Student t-test; Wilcoxon rank sum test |
| Chang et al. 2008 [[82](#_ENREF_82)] | Prospective | Tawain (Chinese) | 92 PDAC | 60 healthy controls | 50 | 57-78 | Serum | VEGF  sVEGF-R1  PIGF | ELISA | Mann-Whitney U-test; Spearman correlation test; Cox proportional hazards model; Kaplan-Meier; Log-rank test |
| Talar-Wojnarowska et al. 2009 [[70](#_ENREF_70)] | Retrospective | Poland | 41 PDAC | 56 CP; 50 healthy controls | 46.3 | 25-47 | Serum | IL-6 | ELISA | Mann-Whitney U-test; Pearson's correlation test |
| Moses et al. 2009 [[56](#_ENREF_56)] | Prospective | Britain (ethnicity not specified) | 42 with unresectable pancreatic cancer | 12 age-matched healthy controls who were admitted to the hospital for benign conditions | 50 | 67 - 71 | Serum | IL-6 | ELISA | Pearson's correlation test; Cox proportional hazards model |
| Bellone et al. 2009 [[71](#_ENREF_71)] | Prospective (2005-2007) | Italy (ethnicity not specified) | 16 PDAC: 1 stage II; 1 stage III; 3 stage Iva; 11 stage Ivb. 8 patients had hepatic metastasis, 1 with lung metastasis, and 2 with peritoneal. 11 patients underwent chemotherapy | Number not specified | 75 | 52 - 83 | PBMC | IL-10; IL-12p40; IL-12p70; IFN-γ | ELISA | Student's t-test; Mann-Whitney test; Kaplan-Meier; Log-rank test |
| Mroczko et al. 2010 [[53](#_ENREF_53)] | Prospective (2003-2006) | Poland | 78 pancreatic cancer (24 underwent tumour resection. 8 stage II, 19 stage III, 51 stage IV) | 70 healthy controls; 45 chronic pancreatitis | 48.7 | 20 - 88 | Serum | IL-6 | ELISA | Mann-Whitney U-test; Kruskal-Wallis test; Kaplan-Meier; Log-rank test; Cox proportional hazards |
| Chen et al. 2010 [[27](#_ENREF_27)] | NA | USA (ethnicity not specified) | 66 pancreatic cancer with stage 2 and 3A | 36 healthy controls; 30 pancreatitis | NA | NA | Serum | MIF | ELISA | Welch's *t* test |
| Vizio et al. 2010 [[80](#_ENREF_80)] | Prospective (January 2006-April 2009) | Italy (ethnicity not specified) | 34 PDAC with advanced or metastatic cancer: 3 stage IIa, 2 stage IIb, 9 stage III, 20 stage IV. All patients received therapy during the study period. 15 underwent surgery | 26 | 55.9 | 47 - 76 | Plasma | VEGF-A; VEGF-D; Ang-1 | ELISA | Student's t-test or Mann-Whitney test; Kaplan-Meier; Log-rank test; Cox proportional hazard regression |
| He et al. 2011 [[112](#_ENREF_112)] | Prospective (5 - 48 month) | Asian (Chinese) | 20 pancreatic cancer (11 stage I-II; 9 stage III-IV) not receiving radiotherapy, chemotherapy, or immune therapy | 15 healthy controls | 67.4 | 43 - 75 | Serum | IL-17; IL-23 | ELISA | Student's t-test; Kaplan-Meier; Log-rank test |
| Ӧzkan et al. 2011 [[47](#_ENREF_47)] |  | Turkey (ethnicity not specified) | 56 PDAC (8 stage I, 10 stage II, 16 stage III, 22 stage 4); 15 peri-ampullary carcinomas; 31 benign pancreatic diseases; 15 benign bile duct and/or gallbladder | 33 healthy controls | 76.1 | 39 - 79 | Serum | MIC-1 | ELISA | Chi-square test; Kruskal-Wallis test; Mann-Whitney U-test; Spearman correlation test |
| Baine et al. 2011 [[19](#_ENREF_19)] | Retrospective | USA (169 Caucasians, 5 African-Americans, 1 Asian, 2 unknown) | 95 PDAC (48 stage I and II, 47 stage III and IV) | 47 healthy controls; 35 chronic pancreatitis | 44.6 |  | PBMC | MIC-1 | multiplex qRT-PCR | Chi-square or Fisher's exact test; ANOVA models; Receiver operation curve analysis |
| Rahbari et al. 2011 [[81](#_ENREF_81)] | Prospective (November 2006 - April 2008) | Germany (ethnicity not specified) | 98 PDAC: 74 with local cancer, 24 with metastatic cancer and 49 receiving adjuvant therapy. | 48 healthy controls; 20 chronic pancreatitis | 50 | 58.7 - 70.7 | Serum | VEGF; VEGFR-1; PIGF; PDGF-AA; PDGF-BB; Ang-1; EGF | ELISA; multiplex protein array | Mann-Whitney test; Spearman's correlation test; Kaplan-Meier; Log-rank test; Cox proportional hazards model |
| Gabitass et al. 2011 [[75](#_ENREF_75)] | Prospective study | United Kingdom (ethnicity not specified) | 46 pancreatic cancer patients | 33 healthy controls; 60 esophagus; 25 gastric | NA | NA | Plasma | IL-2; IL-4; IL-5; IL-6; IL-10; IL-12(p70); IL-13; IL-17; G-CSF; IFN-γ; TNF-α; VEGF | Bio-plex | Wilcoxon rank sum test; Spearman's correlation coefficient; log-rank analysis; Cox proportional hazard. |
| Tjomsland et al. 2011 * | Retrospective | Sweden (ethnicity not specified) | 30 PDAC | 10 healthy controls | NA | NA | Tissue | IL-1α | quantitative PCR (RT-PCR) | Mann-Whitnet test; Kaplan-Meier test; Log rank test |
| Grote et al. 2012 [[42](#_ENREF_42)] | Prospective (1992 - 2006) | 7 European countries | 455 |  | 48.4 | Age at recruitment: 30 - 76; age at diagnosis: 37 - 82 | Serum and plasma | IL-6; sTNF-R1; sTNF-R2 | ELISA | Paired t-test; McNemar's test; Spearman's correlation test; Logistic regression model |
| Sakamoto et al. 2012 [[20](#_ENREF_20)] | Prospective (April 2007 - March 2009 | Japan | 45 PDAC (7 stage I and II, 38 stage III and IV and 23 receiving therapy). 24 had distant metastasis and 23 had lymph node metastasis. | 9 pancreatitis, 16 benign hepatobiliary diseases; 58 colorectal cancer | 61.7 | 31 - 84 | Plasma | GM-CSF; IL-8; VEGF | Antibody suspension bead array | Mann-Whitney test; Cox proportional hazards model; Kaplan-Meier method; log-rank test |
| Vasiliades et al. 2012 [[48](#_ENREF_48)] | Retrospective | Greece (ethnicity not specified) | 30 PDAC; 10 ampullary cancer not receiving therapy | 40 healthy controls | 51.25 | 43 - 80 | Serum | SCF; IL-3; M-CSF; GM-CSF | ELISA | Student t-test; Mann-Whitney test; Spearman's correlstion coefficient; Receiver operation curve analysis; logistic regression models |
| Vizio et al. 2012 [[65](#_ENREF_65)] | Prospective case-control | Italy | 62 PDAC | 20 controls | 62.9 | 31-80 | Plasma | IL-6; IL-17A; IL-23; TGF-β1 | ELISA | Mann-Whitney test; Spearman's correlation coefficient; cox model |
| Chen et al. 2012 [[86](#_ENREF_86)] | Prospective cohort | China | 81 | 40 patients with 15 CP; 8 AP; 8 duodenal adenocarcinoma; 3 gastric carcinoma; 4 colorectalcarcinoma; 2 hepotocellular carcinoma | 48.1 | 45-80 | Tissue and plasma | IL-8 | ELISA; immunohistochemistry | Analysis of variance; Chi square |
| Dima et al. 2012 [[61](#_ENREF_61)] | Prospective | Romania (ethnicity not specified) | 36 PDAC | 22 cancer-free; 9 chronic pancreatitis | 61.1 | 35-74 | Tissue and serum | IL-1β; IL-6; IL-8; IL-10; TNF-α | MILLIPLEX MAP; Immunohistochemistry | Mann-Whitney test; Spearman's correlation coefficient; Cox proportional hazard model and forward stepwise procedures; Contal and O'Quigley method |
| Funamizu et al. 2012* | Retrospective | Germany (ethnicity not specified) | 57 PDAC | Corresponding noncancerous tissues | 47.4 | 38-83 | Tissue | MIF | quantitative PCR (RT-PCR); immunohistochemistry | Kaplan-Meier analysis; Cox proportional hazard regression analysis and stepwise forward and backwards analysis. |
| Ishikawa et al. 2013 [[38](#_ENREF_38)] | Prospective (October 2008 - February 2011) | Japan | 46 PDAC with unresectable cancer receiving adoptive T-cell therapy (40% already received chemotherapy): 11 with locally advanced, 27 with metastatic disease, 8 with recurrent disease. | NR | 47.8 | 39 - 81 | Whole blood | IFN-γ; IL-2; IL-4; IL-5; IL-10; IL-12p70; IL-13; GM-CSF; TNF-α | Flow cytometry | Paired t-test; Cox proportional hazard regression model |
| Kaur et al. 2013 [[52](#_ENREF_52)] | Retrospective | USA, Caucasians; AA; Asian | 91 histologically proven pancreatic cancer | 23 chronic pancreatitis; 24 healthy controls. | 60 | ≥ 18 years | Plasma | MIC-1 | ELISA | Chi square or Fisher exact test; t-tests; ANOVA models; Logistic regression; receiver operation curve analysis |
| Mitsunaga et al. 2013 [[30](#_ENREF_30)] | Prospective (2008 -2011) | Japan | 60 patients with advanced pancreatic cancer patients receiving treatment (gemicitibine monotherapy and GEM-based regimens). | NR | 47 | 35 - 85 | Serum | IL-1β; IL-2; IL-6; IL-8; IL-10; IL-12p40 (IL-12); GM-CSF; IFN-γ; TNF-α | Electro-chemiluminescence assay | Fisher's exact test; Mann-Whitney test; Kaplan-Meier; Log-rank test; Cox proportional hazard regression model |
| Bao et al. 2013 [[43](#_ENREF_43)] | Prospective (1982 - 2008) | USA (Caucasians and African-Americans) | 470 | 1094 | 49.6 | 30 - 84 | Plasma | IL-6; TNF-αR2 | Plasma assay but method not reported | Logistic regression |
| Schultz et al. 2013 [[44](#_ENREF_44)] | Prospective (July 2008 - October 2012) | Denmark and Germany | 559 PDAC: 149 underwent operation (37 of these received adjuvant gemicitabine after operation), 390 with locally advanced or metastatic cancer received treatment. | 318 healthy controls | 57.1 | 31 - 89 | Plasma | IL-6 | ELISA | Spearman's rank correlation; Kaplan-Meier method; Log-rank test; Cox proportional hazards |
| Zhang et al. 2014 [[57](#_ENREF_57)] | Retrospective | China | 163 PDAC; 109 benign pancreatitis.. | 200 healthy controls | 51.9 | 18 - 90 | Serum | GM-CSF; TFN-γ; IL-10; IL-1β; IL-2; IL-4; IL-6; IL-8; TNF-α | Immunoassay | Tukey's multiple comparison test; Metropolis algorithm |
| Breitbart et al. 2014 [[66](#_ENREF_66)] | Prospective (October 2008 - February 2011) | USA (85.1% Caucasians and 8.1% African-Americans) | 43 PDAC with stage III and IV on a stable gemicitabine or chemotherapy. | 32 healthy controls | 53.3 | 28 - 85 | Serum | IFN-γ; TGF-β; TNF-α; IL-1β; IL-2; IL-3; IL-4; IL-5; IL-6; IL-10; IL-12p70 | Meso Scale Discovery technique similar to ELISA | Spearman correlation coefficient; Logistic regression analysis; Wald test. |
| Kahlert et al. 2014 * | Prospective | Heidelberg, Germany ( ethnicity not specified) | 51 PDAC | 44 healthy controls | 56.9 | Median age= 67 years | Serum and tissue | Ang-2; G-CSF; HGF; IL-8; LEP; PDGF-BB; VEGF | Bio-Plex Human Angiogenesis Assay | Pairwise Student t test; Chi squared; Receiver operation curve analysis; Kaplan Meier method; Cox proportional hazards regression model. |
| Blogowski et al. 2014 [[49](#_ENREF_49)] | Retrospective | Poland (ethnicity not specified) | 43 PDAC | 41 healthy controls; 10 pancreatic neuroendocrine tumour; 3 solid pseudopapillary tomours; 31 acute/chronic pancreatitis; 14 pancreatic cyst. | 58.7 | 30 - 70 | Plasma | IL-1; IL-6; IL-8; IL-10; IL-12; IL-17; IL-23; G-CSF; TNF-α | ELISA | Student *t* test; Mann-Whitney test; Pearson or Spearman rank tests, Stepwise multiple regression analysis |
| Shaw et al. 2014 [[50](#_ENREF_50)] | Retrospective | Britain (ethnicity not specified) | 127 PDAC: 20 benign biliary obstruction; 89 with resectable PDAC; 38 with advanced PDAC. | 45 healthy controls; 49 chronic pancreatitis | 68.9 | 29 - 79 | Serum | IL-1β; IL-1ra; IL-6; IL-8; IP-10; MCP-1; MIP-1β; PDGF; Eotaxin | ELISA | Shapiro-Wilk test; Mann-Whitney test; Spearman rank test; Logistic regression analysis; Receiver operation curve analysis ; one-tailed De Long's test; McNennar test. |
| Ren et al. 2014* | Prospective (follow-up: 1-42 months) | Not specified | 44 PDAC | 30 healthy controls; 3 with pancreatic cysts; 15 with early pancreatitis. | 63.6 | 42 - 86 | Plasma | IL-11p | ELISA | Mann-Whitney *U* test; Kruskal-Wallis test; ROC analysis; Kaplan-Meier method; Cox proportional hazards regression model. |
| Torres et al. 2014 * | Prospective | Spain (ethnicity not specified) | 14 PDAC patients who were not received treatment and 13 receiving gemicitabine and erlotinib. 11 with stage III and 28 with stage IV. | 12 healthy controls | 50 | 41 - 79 | Serum | 507 cytokines | Biotin label-based human antibody array | Mann-Whitney test; Receiver operation curve analysis |
| Xu et al. 2014 [[39](#_ENREF_39)] | Retrospective | China | 32 PDAC: 13 with metastasis disease. | 30 | 31.25 | 54 (mean) | Tissue | IL-22 | Western blot analysis; immunohistochemistry | Student *t*-test; Pearson correlation test |
| Komura et al. 2015 [[67](#_ENREF_67)] | Retrospective | Not specified | 20 specimen from PDAC patients | 27 healthy controls | 54.5 | 55-78 | serum | IL-1β, IL-ra, IL-2, IL-4, IL-5, IL-6, IL-7, IL-8, IL-9, IL-10, IL-12 (p70), IL-13, IL-15, IL-17, fibroblast growth factor-basic, eotaxin, G-CSF, IFN-γ, IP-10, MCP-1, MIP-1α, MIP-1β, PDGF-BB, TNF-α, VEGF. | Flow cytometry | Unpaired Student’s *t*-test; Kaplan-Meier method; Log-rank test. |

Ang-1, [angiopoietin 1](https://www.google.co.za/url?sa=t&rct=j&q=&esrc=s&frm=1&source=web&cd=1&cad=rja&uact=8&sqi=2&ved=0CBwQFjAAahUKEwjD88fGrO7GAhWjCNsKHaftD9c&url=https%3A%2F%2Fen.wikipedia.org%2Fwiki%2FAngiopoietin_1&ei=BFivVcPhO6OR7Aan27-4DQ&usg=AFQjCNFUK6ZNd9ckYC6VIVy9gP94Cq0iBg&sig2=xpsj9FNDXQmA4MCPK9-gfA); Ang-2, angiopoietin 2; EGF, epidermal growth factor; G-CSF, granulocyte-colony stimulating factor; GM-CSF, granulocyte-macrophage colony-stimulating factor; HGF, hepatocyte growth factor; IFN-γ, *i*nterferon gamma; IL, interleukin; IL-1F1, interleukin-1F1; IL-1F2, interleukin-1F2; IL-1α, interleukin-1alpha; IL-1β, interleukin-1beta; IP-10, IFN-gamma-inducible protein 10; LEP, leptin; MCP-1, monocyte chemotactic protein-1; M-CSF, macrophage colony-stimulating factor; MIC-1, macrophage inhibitory cytokine-1; MIF, macrophage migration inhibitory factor; MIP-1α, macrophage inflammatory protein 1alpha; MIP-1β, macrophage inflammatory protein 1beta; PD-ECGF, [platelet-derived endothelial cell growth factor](http://www.copewithcytokines.de/cope.cgi?key=Platelet%2dderived%20endothelial%20cell%20growth%20factor); PDGF-AA, platelet-derived growth factor AA*;* PDGF-BB, platelet-derived growth factor BB; PIGF, placenta growth factor; SCF, *s*tem cell factor; TGF, transforming growth factor; TNF, tumor necrosis factor; TNF-R, [tumor necrosis factor receptor](https://www.google.co.za/url?sa=t&rct=j&q=&esrc=s&frm=1&source=web&cd=1&cad=rja&uact=8&ved=0CBwQFjAAahUKEwjsrO-9p-7GAhUyINsKHXefBuM&url=https%3A%2F%2Fen.wikipedia.org%2Fwiki%2FTumor_necrosis_factor_receptor&ei=tFKvVeyCI7LA7Ab3vpqYDg&usg=AFQjCNGL0wgPZeKuRBYBWyKm8OH0k8wx3w&sig2=cTwLYIa4e3lG2gHktTvndw); sTNF-R1, TNF soluble receptors 1; sTNF-R2, TNF soluble receptors 2; VEGF, *v*ascular endothelial growth factor; VEGFR-1, *v*ascular endothelial growth factor receptor 1; NR, not reported; PDAC, pancreatic ductal adenocarcinoma

*References that are not in the manuscript are listed below

***References not in the manuscript**

1. Gansauge F, Gansauge S, Schmidt E, Muller J, Beger HG. Prognostic significance of molecular alterations in human pancreatic carcinoma--an immunohistological study. Langenbecks Arch Surg. 1998 Apr;383(2):152-5. PubMed PMID: 9641888. Epub 1998/06/26. eng. doi: 10.1016/S0304-3835(98)00259-6

2. Funamizu N, Hu C, Lacy C, Schetter A, Zhang G, He P, et al. Macrophage migration inhibitory factor induces epithelial to mesenchymal transition, enhances tumor aggressiveness and predicts clinical outcome in resected pancreatic ductal adenocarcinoma. Int J Cancer. 2012 Jul 23. PubMed PMID: 22821831. Pubmed Central PMCID: PMC3488363. Epub 2012/07/24. Eng. doi: 10.1002/ijc.27736.

3. Kahlert C, Fiala M, Musso G, Halama N, Keim S, Mazzone M, et al. Prognostic impact of a compartment-specific angiogenic marker profile in patients with pancreatic cancer. Oncotarget. 2014 Dec 30;5(24):12978-89. PubMed PMID: 25483099. Pubmed Central PMCID: PMC4350362. Epub 2014/12/09. eng. **doi: 10.18632/oncotarget.2651**

4. Ren C, Chen Y, Han C, Fu D, Chen H. Plasma interleukin-11 (IL-11) levels have diagnostic and prognostic roles in patients with pancreatic cancer. Tumour biology : the journal of the International Society for Oncodevelopmental Biology and Medicine. 2014 Nov;35(11):11467-72. PubMed PMID: 25123265. Epub 2014/08/16. eng. doi: 10.1007/s13277-014-2459-y.

5. Tjomsland V, Spangeus A, Valila J, Sandstrom P, Borch K, Druid H, et al. Interleukin 1alpha sustains the expression of inflammatory factors in human pancreatic cancer microenvironment by targeting cancer-associated fibroblasts. Neoplasia (New York, NY). 2011 Aug;13(8):664-75. PubMed PMID: 21847358. Pubmed Central PMCID: PMC3156657. Epub 2011/08/19. eng. DOI: http://dx.doi.org/10.1593/neo.11332

6. Torres C, Perales S, Alejandre MJ, Iglesias J, Palomino RJ, Martin M, et al. Serum cytokine profile in patients with pancreatic cancer. Pancreas. 2014 Oct;43(7):1042-9. PubMed PMID: 24979617. Epub 2014/07/01. eng. doi: 10.1097/MPA.0000000000000155.
